# Within-host microbial selection and multiple microbial generations buffer the loss of host fitness under environmental change

**DOI:** 10.1093/femsec/fiaf089

**Published:** 2025-09-08

**Authors:** William S Pearman, Allen G Rodrigo, Anna W Santure

**Affiliations:** University of Auckland, School of Biological Sciences, 3A Symonds Street, Auckland 1142, New Zealand; Centre for Computational Evolution (CCE), Faculty of Science, University of Auckland, Auckland 1142, New Zealand; University of Auckland, School of Biological Sciences, 3A Symonds Street, Auckland 1142, New Zealand; Centre for Computational Evolution (CCE), Faculty of Science, University of Auckland, Auckland 1142, New Zealand; University of Auckland, School of Biological Sciences, 3A Symonds Street, Auckland 1142, New Zealand; Centre for Computational Evolution (CCE), Faculty of Science, University of Auckland, Auckland 1142, New Zealand

**Keywords:** computational model, evolution, holobiont, host-microbiome interactions, microbiome, selection, simulation

## Abstract

The relationship between, and joint selection on, a host and its microbes—the holobiont—can impact evolutionary and ecological outcomes of the host and its microbial community. We develop an agent-based modelling framework for understanding the ecological dynamics of hosts and their microbiomes. Our model incorporates numerous microbial generations per host generation allowing selection on both host and microbes. We then explore host and microbiome fitness and diversity in response to environmental change. We demonstrate that multiple microbial generations can buffer changes experienced across host lifetimes by smoothing environmental transitions. Our simulations reveal that microbial fitness and host fitness are at odds with each other when considering the impact of vertical inheritance of microbial communities from a host to its offspring—where high parent-offspring microbial transmission favours microbial fitness, while low transmission favours host fitness. These tradeoffs are minimized when microbial generation count per host generation is high. This may arise from ‘cross-generational priority effects’ which maintain diversity within the community and can subsequently enable selection of beneficial microbes by the host. Our model is extensible into new areas of holobiont research and provides novel insights into holobiont evolution under variable environmental conditions.

## Teaser

Our model demonstrates that multiple microbial generations per host generation are important for allowing hosts and microbes to co-evolve and to buffer against environmental change.

## Introduction

Over recent years there has been an increasing interest in understanding host-microbiome co-evolution and the potential for the formation of a ‘holobiont’ (Zeng et al. 2015, Henry et al. 2021, Bruijning et al. 2022, Roughgarden 2023). This interest has been largely prompted by the increasing awareness of the role of microbiomes in influencing host biology, and the idea that microbes, as a result of their shorter generation times, may adapt faster to changing environments and in turn facilitate host adaptation to these changing environments (Petersen et al. 2023, Shah et al. 2024). Although the term ‘holobiont’ is somewhat loaded and complex, with a short but controversial history (Moran and Sloan 2015), it is generally used to describe a host and its microbiome as a joint unit upon which natural selection can act (Rodrigo 2023). Because the microbiome can play a role in shaping host fitness (Henry et al. 2021), there is the potential for multilevel selection wherein natural selection operates on the microbiome, the host, and the joint ‘unit’ (van Vliet and Doebeli 2019). This idea of multi-level selection with regards to the holobiont has been well modelled through a range of different approaches, with notable advances made by Zeng et al. (2015, 2017), van Vliet and Doebeli (2019), Roughgarden (2023), and Bruijning et al. (2022). These works largely build off each other and serve to provide a strong theoretical framework for understanding holobiont evolution and ecology.

Holobiont theory is often centred around the mode of transmission of microbes, or where a host obtains their microbiome from. This is understandably of notable importance, as traditional or neo-Darwinian evolutionary theory anticipates a lineal inheritance pattern for the evolution of traits. This ‘lineal’ inheritance is referred to as vertical transmission, and contrasts with horizontal transmission, which is the acquisition of microbes from other sources (most commonly the environment). However, ‘mixed mode transmission’, where hosts acquire some proportion of their microbiome via vertical transmission, and the remainder from the environment, is likely the dominant form of microbial acquisition (Ebert 2013). Other important sources of microbial transmission can be through social interactions of hosts (Sarkar et al. 2024) or through indirect non-lineal inheritance of microbes whereby microbes shed from hosts into the environment are then acquired by other hosts within the environment. The modes of microbial acquisition by hosts can influence the type and scale of host adaptation, e.g. strict vertical inheritance can perhaps lead to long-term host-microbe partnerships, enabling hosts to outsource functions to their microbiome (with microbes evolving altruistic traits)—resulting in a fitness benefit to the host (van Vliet and Doebeli 2019). Alternatively, strictly horizontal acquisition can provide shorter-term ecological adaptations of the host to the environment and enable ‘switching’ of microbes to more favourable ones where necessary (Petersen et al. 2023).

In this paper, we consider a holobiont as an ecological unit which participates in the evolutionary process, but is not *sensu stricto* a unit of selection (see Roughgarden 2020, 2023, Suárez and Stencel 2020, Veigl et al. 2022 for comprehensive discussions of the holobiont’s participation in the evolutionary process). In our case we define a holobiont as the relationship between a macroorganism and a community of microbes whereby both host and microbe fitness are impacted by their relationship, and selection can operate on host, microbe, and their relationship. Finally, although previous agent-based model approaches do not necessarily refer to their systems as holobionts (Zeng et al. 2017, Bruijning et al. 2022), there is an implicit argument that this is what they are modelling (Rodrigo 2023). Thus, we prefer to be explicit in what we model and how we define a holobiont.

As noted by Rodrigo (2023), there are three broad types of models of holobiont evolution. The first, by van Vliet and Doebeli (2019) makes use of multilevel selection theory to understand holobiont evolution and is expressed as a set of mathematical equations rather than as an agent-based process. van Vliet and Doebeli (2019) suggest that microbes can evolve altruistic traits under short host-generation times and high levels of vertical inheritance—even when there is a cost to the microbe. Counter to this, the second type of model from Roughgarden (2020) and (2023) propose that a ‘collective’ inheritance of microbes from the environment can lead to the formation of a holobiont through host filtering/selection for environmental microbes, suggesting that vertical transmission is not essential for holobiont formation. Finally, the last set of models are agent-based processes first pioneered by Zeng et al., (2015, 2017, 2018) with commonalities with those models of Bruijning et al. (2022) and Daybog and Kolodny (2023). Zeng et al. (2015, 2017) developed a model which includes both host and microbial selection, this work showed that host selection alone is not sufficient to suppress microbial diversity, while microbial selection can suppress microbial diversity alone. The model developed by Zeng has been subsequently extended by Bruijning et al. (2022) and Daybog and Kolodny (2023). Bruijning et al. (2022) demonstrate that the fitness benefits to a host from vertical transmission are dependent on both the predictability and variance of an environment. Daybog and Kolodny (2023) demonstrated in their version of the model that factors such as population size and microbial community richness can enable high microbial beta diversity (i.e. large differences in the microbial communities between hosts). This increased beta diversity occurs despite the intuition that, in contrast to neutral scenarios, selection should drive the host-associated microbiome to an ‘optimal’ composition—thus if the microbiome is a ‘trait’ under selection, then reduced diversity may be expected.

Our work has been prompted by the observation that there are three core assumptions regularly made with regards to holobiont simulations. The first is that of partial neutrality, where microbial communities are neutrally assembled within the host without consideration of selection operating on the microbes directly—in essence, microbes within a host are ‘equally fit’ under this scenario but their contribution to host fitness varies, thus selection acts indirectly on the microbiome as a result of host reproduction (Zeng et al. 2017, Bruijning et al. 2022, Daybog and Kolodny 2023). The second assumption often made is that the environmental community is relatively simple—composed of the previous host generation's combined microbiomes, which results in a ‘collective’ inheritance from one host generation to another (Roughgarden 2020, Daybog and Kolodny 2023). Alternatively, the environmental pool may be made up of a ‘fixed’ unchanging pool of equally abundant microbes (Bruijning et al. 2022), or a combination of both approaches (Zeng and Rodrigo 2018). In reality, environments are likely to have a substantial proportion of resident microbes that are themselves reproducing and subject to selection. For example, in an estuary the microbial pool is likely to be comprised of a fairly constant input of upstream microbes (i.e. a fixed environmental pool, following Zeng et al. 2015, 2017; Bruijning et al. 2022), the ‘shedding’ of microbes from hosts into the environment (Zeng et al. 2015, Bruijning et al. 2022, Daybog and Kolodny 2023), and reproduction of microbes that are already within the environment. Ultimately, we aimed to provide an environmental microbial population which is governed by immigration (i.e. from the fixed environment), proliferation (reproduction of microbes within the environment), and shedding (from hosts already present) in order to reflect known ecological processes (Leung and Lee 2016, Albright and Martiny 2018).

The third common assumption is that there are one or few microbial generations per host generation. Indeed, the assumption regarding one microbial generation per host generation, despite being practically useful for simulations, ignores an oft-cited argument for the benefits of a microbiome—that multiple microbial generations per host generation can facilitate adaptation of the host in changing environments (Ferreiro et al. 2018, Kolodny and Schulenburg 2020). Bruijning et al. (2022) simulate up to eight microbial generations within a host generation—yet assume that microbial communities are neutrally assembled within the host, thus for selection to operate on the microbiome, it must be indirect through host reproduction. Making such assumptions can be advantageous, both with regards to simplicity but also with regards to computational feasibility (e.g. Munoz et al. 2018). However, jointly considering the role of within-host selection and multiple microbial generations is an important step towards understanding holobiont evolution (Sieber et al. 2019), particularly because the fitness of both hosts and microbes must benefit from the relationship (or at least not be negatively affected) in order for a host-microbiome pair to evolve jointly as a holobiont evolutionary unit.

Here, we develop a model framework as an extension of work by Bruijning et al. (2022) and Zeng et al. (2017) to explore how host and microbe fitness interact with each other under selection upon both the host and the microbes directly, multiple microbial generations per host generation, and multiple microbial sources. Microbes are acquired by the host from their parents, and from an environmental pool that is comprised of a fixed environmental pool, shedding of microbes from hosts in the environment, and—the new component we incorporate into our model—an additional environmental microbial contribution from an autochthonous source, i.e. a pool of microbes existing in the environment. Our model also allows for environmental variation to occur at the microbial timescale, allowing for more realistic community dynamics. This is particularly important when multiple microbial generations per host generation are simulated, because microbes reproduce much faster than their hosts and thus experience environmental change at a finer timescale than that of their hosts.

Our objectives here are two-fold—first, we wish to determine the influence of both multiple microbial generations per host generation and within-host selection on microbial alpha diversity (i.e. the diversity of microbes within each host, both the number of unique species and their relative abundances). Secondly, we aim to explore this question in the context of various types of changing environment (i.e. predictable vs. unpredictable environments, increasing variance, or increasing mean environmental conditions), as an oft-cited justification for holobiont research is that the microbiome could facilitate host adaptation to changing environments (Baldassarre et al. 2022, Shah et al. 2024). Finally, although not expounded upon in this work, we aim to provide a framework with sufficient flexibility to accommodate future work regarding the interactions between host genetics and the microbiome in shaping host evolution.

## Materials and methods

### Modelling framework

Our model establishes a constant-sized population (*N_h_* = 100) of haploid hosts. A number (*N_m_* = 200) of microbial taxa make up large microbial communities present within each host (of population size *n_mh_* = 10^6^) and in an external environment (of population size *n_mE_* = 10^8^). Both hosts and microbial taxa are assigned trait values (*φ_h_* and *φ_m_*, respectively) that determine their fitness. Host trait values are determined by both their own initial genetic trait value (*φ_hg_* —here fixed at 0) with weighting *G*, and by the mean trait value of their microbiome (*φ_hM_*), with weighting (1-*G*). Our framework is intentionally broad, and it includes many parameters and variables which are not explored fully in this work—instead, we develop the terminology to guide a broader modelling framework, and present the results of a simplified version of the model. In the present simulations, by fixing *φ_hg_* at 0, the hosts have no adaptive potential without a microbiome, as there is no genetic variation within the population. Trait values for each microbial taxon are drawn from a uniform distribution between −2.5 and 2.5, with the range corresponding to minimum and maximum environmental values simulated in our model. The difference between the environmental value and the trait value of a host or microbe determines its fitness in the given environmental condition, in accordance with assumptions of stabilizing selection. For each host generation, there are a fixed number of microbial generations both within the host and in the external environment (*T_M_* ≥ 1). At each generation, whether host or microbe, the communities are entirely replaced, as we do not model overlapping generations, and both hosts and microbes reproduce clonally.

#### Microbial inheritance and acquisition

**Figure 1.**
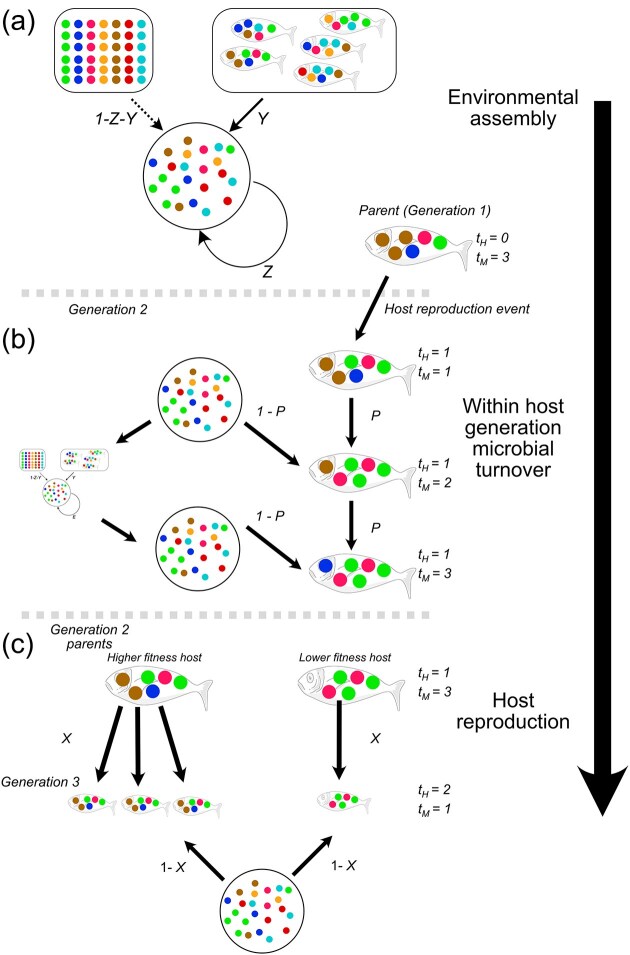
Diagram of simulation process. Arrows within each panel represent multinomial sampling events based on the product of fitness and relative abundance. In (a) the environmental microbial pool is assembled based on some contribution of its previous state (*Z*), some contribution from the hosts (*Y*), and the remainder from a fixed environmental pool (1-*Z*-*Y*). (b) Host microbes undergo change for a specific number of microbial generations (in this example, 3), where the environment also undergoes change during this time. Host microbes are sampled at each microbial generation from the host with proportion *P* and from the external environment with proportion 1-*P*. (c) Hosts undergo reproduction where their fitness is determined based on their current microbiome, their offspring inherit a proportion *X* of their microbiome from their parent, with the remainder being made up of colonizers from the environment. All sampling events in solid arrows indicate fitness-based multinomial sampling, with dashed lines indicating random sampling.

The simulation begins with an environmental pool initially comprised of all microbial taxa at approximately equal abundances (with the absolute count of each microbe sampled multinomially, with each microbe having the same sampling probability following Zeng et al. 2015, 2017), and the first generation of hosts acquire their microbiome multinomially from this pool with each microbe having equal sampling probability. All subsequent generations are subject to fitness-based selection. The environmental pool is then renewed upon each microbial generation and is made up of some proportion, *Y*, being shed from the collective host microbial pool (thus accounting for non-lineal inheritance of microbes), some proportion *Z* being made up from the previous environmental pool, and a final proportion (1-*Z*-*Y*) being made up from the fixed environment with all microbial taxa at equal abundances (Fig. 1a). Microbial taxa are sampled multinomially from each source until the total number of microbes is reached, where the probability of being sampled is the product of the fitness and the relative abundance of the microbial taxa within the relevant source. Following Turelli (1984), we assume stabilizing Gaussian selection towards the environmental condition ${{E}_{{{t}_M}}}$ and therefore calculate the fitness of microbial taxon *m* in the environment as:


\begin{eqnarray*}
{{\omega }_{m,env}} = \ {{e}^{ - \frac{{{{{\left( {{{\varphi }_m}\ - {{E}_{{{t}_M}}}} \right)}}^2}}}{s}}}
\end{eqnarray*}

where ${{\varphi }_m}$ is the trait value for a specific microbe, the environmental condition ${{E}_{{{t}_M}}}$ corresponds to the trait value that maximizes microbe fitness in the environment at each microbial timepoint (${{t}_M}$) and *s* > 0 is the selection parameter (set at 1 for this work) that determines fitness, here related to the strength of selection on the microbe in the environment, with small values of *s* corresponding to stronger selection and very large values approaching neutrality. The environmental condition is allowed to vary over time (discussed in detail below).

Because there may be many generations of microbes within a host generation, the microbiome can change over the lifetime of the host (Fig. 1b). With each microbial generation within the host, the microbiome is sampled from its previous generation of that host at proportion *P*, and from the environment at proportion 1-*P*. Our model thus assumes that microbial reproduction occurs at the same timescale as microbial acquisition by hosts and is uniform across all microbes (see Roughgarden 2023 for an alternative approach). As per the strategy within the environment, microbial taxa within the host are sampled multinomially from each of the now two sources (the environment and the host), where the probability of being sampled is the product of the fitness and the relative abundance of the microbial taxa in that source. The fitness of microbial taxon *m* within a host *h* is calculated as:


\begin{eqnarray*}
{{\omega }_{m,\ \textit{host}}} = {{e}^{ - \frac{{{{{\left( {{{\varphi }_m}\ - \ {{E}_{{{t}_{Mh}}}}} \right)}}^2}}}{s}}}
\end{eqnarray*}

where the within-host environment (${{E}_{{{t}_{Mh}}}}$) is determined as follows:


\begin{eqnarray*}
{{E}_{{{t}_{Mh}}}} = I{{\varphi }_{hg}} + \left( {1 - I} \right){{E}_{{{t}_M}}}
\end{eqnarray*}

where *I* is the weighting of the host genetic trait and (1-*I*) the weighting of the current environmental condition (${{E}_{{{t}_M}}}$), and *s* > 0 is again the selection parameter (set at 1 for this work), here representing selection of the microbe within the host. Note that the weightings *I* and (1-*I*) are chosen to reflect the influence of the internal host environment and external environment on a host's microbiome. Such an approach to weighting assumes that host and microbial phenotypes are on the same scale as each other and the external environmental condition, this is necessary in models such as ours which utilize models of stabilizing selection in which maximal fitness is achieved by a phenotype that matches the environment (Nuismer et al. 2013). A biological analogue of this approach to weighting would be a skin vs. gut microbiome, where the skin microbiome is far more influenced by the external environment due to direct exposure—conversely, a gut microbiome is more influenced by host-specific factors such as diet, gut pH, or temperature (Reese and Dunn 2018, Webster et al. 2020, Woodhams et al. 2020). Furthermore, there is extensive literature demonstrating that locally adapted microbes can contribute to host fitness, in a similar way to that which we present in our model (see Henry et al. 2021 for a review of this). The results we present principally correspond to *I* = 0.5, representing an equal contribution from the host and environment on the microbe's fitness, but see Supplementary Fig. 1 and Supplementary Fig. 2 for an exploration of various values of *I*.

#### Host reproduction and selection

After the specified number of microbial generations, *T_M_*, has passed, the host undergoes fitness-based reproduction (Fig. 1c), where hosts are sampled with replacement with a probability equal to their fitness. Host phenotypes are first calculated as:


\begin{eqnarray*}
{{\varphi }_h} = G{{\varphi }_{hg}} + \left( {1 - G} \right){{\varphi }_{hM}}
\end{eqnarray*}

Where ${{\varphi }_{hM}}$ is the mean microbial trait value of a host’s microbiome, *G* is the weighting of host genetics relative to the microbiome (fixed at 0 in this work except when comparing to no host reliance on the microbiome), and ${{\varphi }_{hg}}$ is the heritable host genetic trait (fixed at 0 in this work). Host fitness is then calculated from its phenotype as:


\begin{eqnarray*}
{{\omega }_{h,\ env}} = {{e}^{ - \frac{{{{{\left( {{{\varphi }_h} - \ {{E}_{{{t}_H}}}} \right)}}^2}}}{s}}}
\end{eqnarray*}

where ${{E}_{{{t}_H}}}$ is the environmental condition at that timepoint and thus is also the trait value that maximizes host fitness in the environment at each host timepoint (${{t}_H}$) and *s* is again the selection parameter (set at 1 for this work).

Following host reproduction, offspring acquire a new microbiome with some proportion, *X*, being sampled from their parent and the remainder (1-*X*) from the environment. Microbial taxa are sampled multinomially from each source until the total number of microbes is reached, where the probability of being sampled is again the product of the fitness and the relative abundance of the microbial taxa within the relevant source. In essence, this creates two forms of horizontal transmission of microbes—the transmission of microbes into a host at birth (in our model this is 1-*X*), and the transmission of microbes over a host’s lifetime (1-*P*) (Robinson et al. 2019). A description of all parameters available in our framework, and the values used for the presented work, is available in Table 1. We also provide fully annotated code for reproducing our agent-based model, along with the analyses and simulations presented herein, at https://wpearman1996.github.io/Holobiont_Bookdown/.

**Table 1.** Description of parameters available in our host-microbiome modelling framework, and the choice of values specifically used in the presented simulations.

Term/parameters	Description	Values in simulations presented in the main text
*N_H_*	Number of hosts	100
*N_M_*	Number of microbial taxa	200
*n_MH_*	Number of microbes within each host	10^6^
*n_ME_*	Number of microbes within the environment	10^8^
*T_M_*	Number of microbial generations per host generation. Lower case *t* refers to specific time points	1, 2, 3, 4, 5, 6, 7, 8, 9, 10, 20, 50, 100, 200
*T_H_*	Number of host generations. Lower case *t* refers to specific time points	1500
*φ_hg_*	Heritable host genetic trait value	0
*φ_m_*	The trait value for a microbial taxon	Varies across taxa between -2.5 and 2.5
*φ_hM_*	Mean trait value of a host’s microbiome	Determined by microbiome community per host
*G*	Weighting of host genetic trait value to its overall host trait value.	0, 1
1-*G*	Weighting of mean microbial trait value to host trait value.	Determined by *G* above
*φ_h_*	Host phenotype, calculated as an average of *φ_hg_* and *φ_hM_* weighted by *G*.	Variable—depends on G.
*Z*	Contribution of previous microbial generation to current environmental microbial pool.	0.8*
*Y*	Joint contribution of the previous generation of host-microbiomes to subsequent environmental microbial pool	0.05
1-*Y-Z*	Contribution of fixed, invariant, microbial pool to subsequent environmental microbial pool.	0.15*
*X*	The contribution of a parent to its offspring’s microbiome (i.e. vertical inheritance).	0, 0.167, 0.333, 0.500, 0.667, 0.933, 0.990, 0.999, 1
1-*X*	The contribution of the environment, rather than a parent, to a host microbiome (i.e. horizontal transmission).	Determined by *X* above
*P*	The proportion of a host's microbiome that contributes to the subsequent microbial generation within a host generation.	0.98
1-*P*	The proportion of a host's microbiome that is acquired from the environment for each microbial generation within a host generation.	0.02
*E*	Environmental condition; ${{E}_{{{t}_M}}}$ is the trait value that maximizes microbe fitness in the environment at each microbial timepoint (*t_M_*) while ${{E}_{{{t}_H}}}$ is the host trait value that maximizes host fitness in the environment at each host timepoint (*t_H_*). ${{E}_{{{t}_H}}}$ = ${{E}_{{{t}_M}}}$when *T_M_ =1*.	Specific to each scenario.
${{E}_{{{t}_{Mh}}}}$	The composite environment which imposes selection upon the host- associated microbe, and is a weighted average of the host genetic trait (*φ_hg_)* and ${{E}_{{{t}_M}}}$	
*s*	The strength of selection. Smaller values indicate stronger selection, while very large values approach neutrality.	1
*I*	Relative contribution of the internal environment, as defined by the host genetic trait value (*φ_hg_*), to shaping the environmental condition for microbes within the host (${{E}_{{{t}_{Mh}}}})$. High values mean greater influence of *φ_hg_* on ${{E}_{{{t}_{Mh}}}}$.	0.5*
1-*I*	Relative contribution of the external environment to shaping the environmental condition for microbes within the host.	0.5*

* indicate cases where alternative values for these parameters have been explored; see Supplementary Information

### Influence of changes in environmental conditions on host and microbe fitness and microbial diversity

**Figure 2.**
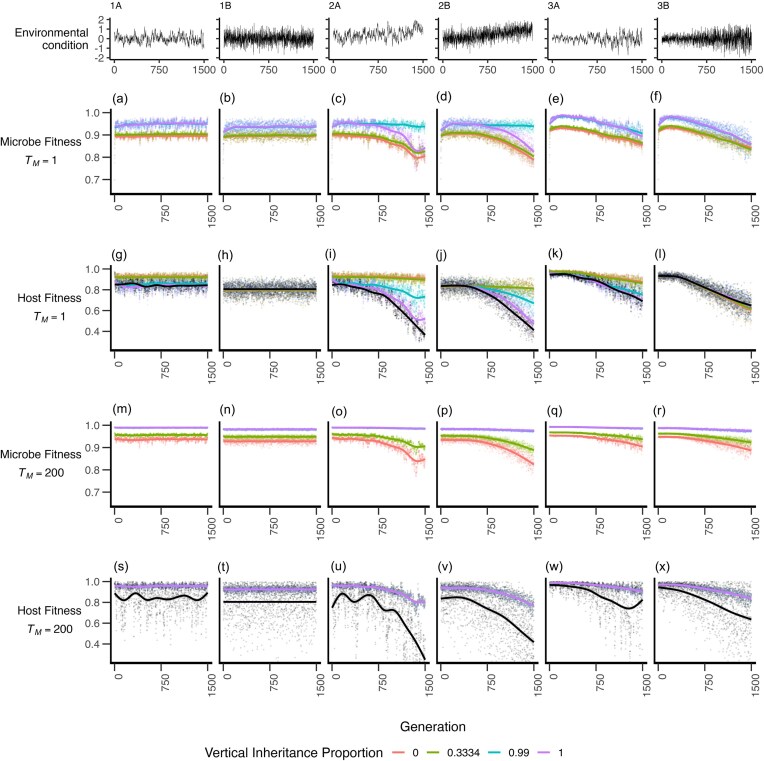
Microbial fitness within the host (a–f) and host fitness (g–l) over the course of the simulations when the number of microbial generations per host generation (T_M_) is 1. Microbial fitness within the host (m–r) and host fitness (s–x) over the course of the simulations when the number of microbial generations per host generation is 200. Lines represent GAM smoothing; data represent mean values from 20 replicate simulations where *G* = 0 (i.e. host fitness is determined only by its microbiome and not by its own genetic value). Black lines in panels g–l and panels s–x are the host fitness where the contribution of the microbiome to host phenotype is zero (i.e. *G* = 1), this value does not affect microbial fitness and as such is not displayed on panels a–f or m–r. Environmental scenarios span no mean change to the environment (panels 1A and 1B), increasing mean environment (2A, 2B), increasing variation in the environment (3A, 3B), and variation introduced by autocorrelated and random variation (A and B, respectively).

We explore the influence of environmental conditions, the fitness of both the host and microbe, and the diversity of the microbial community within each host, while varying the number of microbial generations per host generation (*T_M_*) and the proportion of vertical (*X*) versus horizontal inheritance (1-*X*) of microbial taxa at the point hosts reproduce. Our approach is in part an extension of Bruijning et al., (2022); however, it differs in that we simulate microbe fitness alongside host fitness rather than relying on neutral processes for the microbes. Therefore, to understand how host and microbe fitness interact, we produced six scenarios of different types of changing environments exemplified in Fig. 2—panels 1A–3B. All scenarios had an initial burn-in period of 200 host generations to allow for microbial communities to stabilize following initialization (Supplementary Fig. 3). This value was chosen as it is the anticipated number of generations required for all members of the population to share a common ancestor (i.e. the number of generations, 2*N_e_*, for the host population to reach coalescence).

In short, we produce (1) ‘unchanging’ environments, where the environment fluctuates around a mean, creating variation around that mean, (2) an increasing mean environment where the mean environmental condition increases over time—with variation around this changing mean, and finally (3) an environment where the variance around the mean increases over time but the mean itself does not. For each of these scenarios we have two versions: (A) a high autocorrelation version where the environmental conditions from each point to the subsequent are highly autocorrelated or (B) a ‘random’ variation version where the environmental condition is drawn independently of the previous time point (see Supplementary Information for detailed methods for generation of environmental conditions). The exploration of the effect of autocorrelation was motivated by the need to better understand the impact of the anticipated increases in environmental autocorrelation under climate change on the host and microbial communities, in addition to also recognizing that environmental conditions are not usually ‘random’ but instead have some degree of autocorrelation (Vasseur et al. 2014, van der Bolt et al. 2018). Autocorrelation in this instance essentially means how predictable the subsequent environmental condition is based on the preceding condition—highly autocorrelated thus means highly predictable. An effect of high autocorrelation is that when environmental transitions do occur, they are likely to be more severe and longer lasting, in reality, this can be manifested by events such as extended heat waves (Di Cecco and Gouhier 2018).

In our simulations, we generated 20 independent replicates of each environmental condition, with an environmental value ${{E}_{{{T}_H}}}$ generated for each host generation (here, *T_H_* = 1500). These environmental conditions were then reused for each combination of the number of microbial generations per host generation (*T_M_*) and the proportion of vertical (*X*) versus horizontal inheritance (1-*X*) (see below)—this means across all simulations there were 120 unique sets of ${{E}_{{{T}_H}}}$ (6 environmental conditions, 20 replicates). For each value of ${{t}_M}$, the environmental conditions between subsequent host timepoints ${{t}_H}$ were linearly interpolated *T_M_* times. For example, if ${{E}_{{{t}_H}}}$ at ${{t}_H}$  *=* 1 is 0.2 and ${{t}_H}$  *=* 2 is 0.24, and *T_M_* = 4, then the values for ${{E}_{{{t}_M}}}$ would be {0.2,0.21,0.22,0.23}. When the number of microbial generations per host generation is 1, ${{E}_{{{t}_H}}}$ = ${{E}_{{{t}_M}}}$.

**Figure 3.**
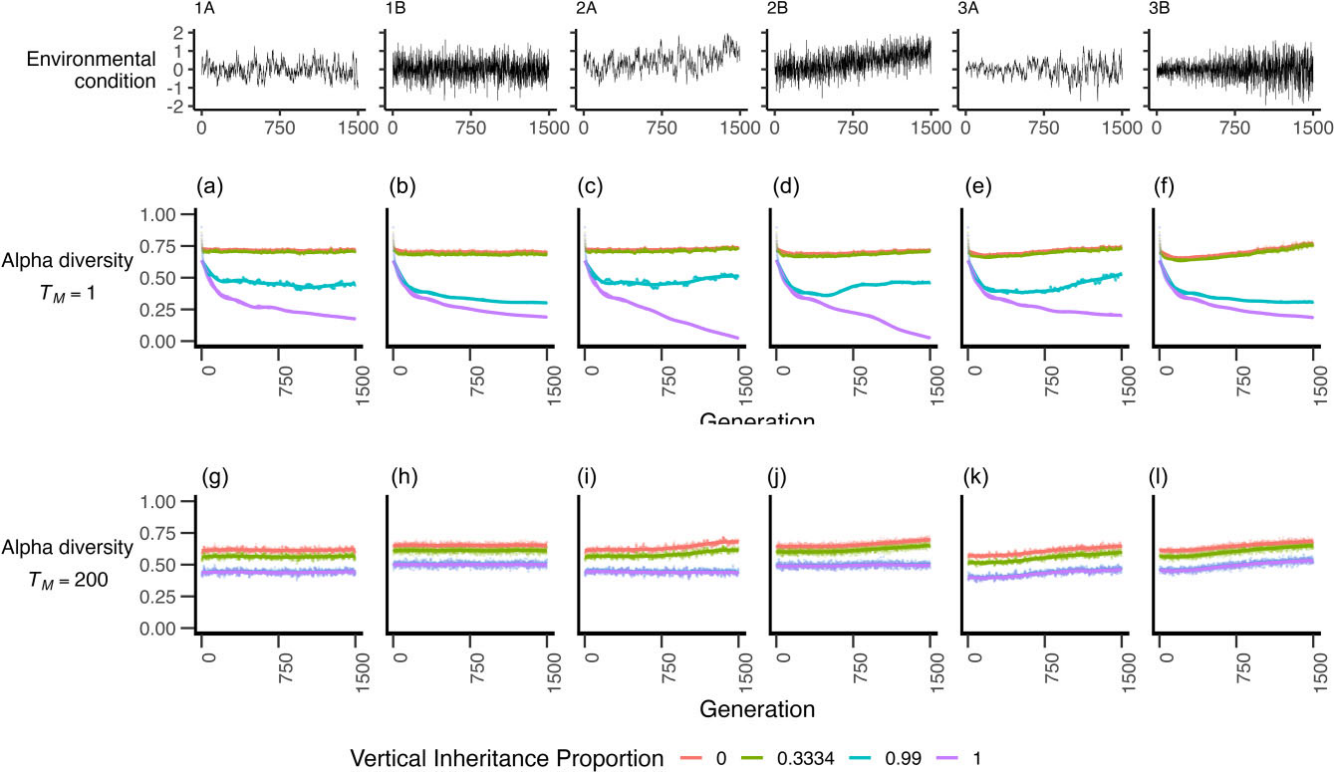
Alpha diversity of microbiomes at host generation 1500 in response to both vertical inheritance proportion and number of microbial generations per host generation (a-f), alpha diversity over the course of the simulations when the number of microbial generations per host generation (T_M_) is 1, and (g-l) over the course of the simulations when the number of microbial generations per host generation is 200. Lines represent GAM smoothing; data represents mean values from 20 replicate simulations. Environmental scenarios 1A–3B are as described in Fig. 2.

Our simulations covered a range of microbial generations per host generation (*T_M_* = 1, 2, 3, 4, 5, 6, 7, 8, 9, 10, 20, 50, 100, 200) and a range of vertical inheritance at host reproduction (*X* = 0, 0.167, 0.333, 0.500, 0.667, 0.933, 0.990, 0.999, 1). In all simulations, the overall host trait value is determined solely by its microbial population (with the exception of the black lines in Figs 2 and 3, where *G* = 1). The selection parameter was set to 1, corresponding to medium-strong selection on both hosts and microbes (see Supplementary Fig. S4 for visualization of fitness landscape under different values of *s*). The value of *s* was chosen based on both its use within the literature (Johnson and Barton 2005, Clo 2022) and to make selection strong enough to overcome the effects of drift within our relatively small host population. We set *P* = 0.98 such that 2% of microbes within the host are acquired externally from the environment compared to the previous microbial pool in the host. Finally, *Z* = 0.8 and *Y* = 0.05 were set to establish the contributions of the current environmental microbial community, host shedding of microbes, and a fixed microbial input to the environmental microbial community (consisting of 10^8^ microbes). See Supplementary Fig. S5 for an exploration of various values of *Z*.

For each simulation, at the end of every host generation (i.e. prior to reproduction), we recorded the average host fitness and average microbial fitness within the host, as defined by the equations above. Further, we recorded average microbial alpha diversity across hosts for each replicate host as the scaled Shannon-Weiner index following Zeng et al. (2015).

## Results

We first present case studies where the number of microbial generations per host generation (*T_M_*) is either 1 or 200 across different environments and levels of vertical inheritance (*X*) in order to describe changes in fitness and diversity over the course of the simulations. The environmental trajectories in our simulations were either highly autocorrelated (type A) or random (type B), under scenarios where either there was no net change over time (scenario 1), an increasing mean environmental condition (scenario 2), or increasing variance without a net change (scenario 3). We then present fitness and diversity results at the end of each simulation, so that the impact on these metrics across the range of values of *T_M_* can be explored.

### Dynamics of microbe and host fitness, one microbial generation per host generation

We first explored the changes in microbial and host fitness over time across environmental conditions when there was only one microbial generation per host generation. Beyond the initial stabilization of the communities in the 200 generations, both host and microbial fitness were largely unchanging in a variable environment that did not change systematically over time, whether environmental conditions were autocorrelated or random (Fig. 2a, b, g, h).

In the net environmental change scenario (Fig. 2—panels 2A, 2B) we found that microbial fitness declined over time, with larger declines observed with lower vertical inheritance (Fig. 2c, d, Supplementary Fig. S5c, d). Conversely, these low levels of vertical inheritance tended to maximize host fitness (Fig. 2i, j, Supplementary Fig. S6 i, j). In cases of increasing variation (Fig. 2—panels 3A, 3B), both host and microbe fitness declined over time (Fig. 2e, f, k, i).

In most instances, the host fitness when derived from the microbiome was higher than that of the fitness when derived solely from a host's trait value (with the exception of extremely high vertical inheritance in highly autocorrelated environments—Fig. 2g, i, k).

In addition to the relationships with environmental conditions, we found that generally the degree of vertical inheritance that maximized host fitness tended to minimize microbial fitness—essentially resulting in antagonistic fitness between host and microbe when the microbial generations per host generation (*T_M_*) was 1. More explicitly, high levels of vertical inheritance appeared to lead to higher microbial fitness, but lower host fitness. The exception to this was where vertical inheritance was complete, in this scenario both microbial and host fitness tended to decline below that of the 99% vertical inheritance scenario. Such an outcome is, however, anticipated—there are no chances to recruit new microbes when vertical inheritance is 100% and the microbial generation count is 1. Thus, a burn-in period of 200 generations results in selection for microbes suited to the burn-in environment—subsequent changes in the environment over time then force the loss of these microbes, but without a chance to replace them with ‘fitter’ microbes. This then has a flow-on effect to host fitness, which in our model is derived solely from the microbiome. As a result, we found that complete vertical inheritance when *T_M_* = 1, tended to result in near-equivalent fitness to a simulation where hosts derived their fitness entirely from their own genotype rather than entirely from the microbiome. Beyond this, we found that any level of vertical inheritance < 1 resulted in higher host fitness (Fig. 2s–x).

In addition, we typically found that host fitness was higher under autocorrelated environmental conditions, especially when vertical inheritance was low (Supplementary Fig. S7a–c). This difference was increasingly pronounced under the increasing variance environmental scenario.

### Dynamics of microbe and host fitness–200 microbial generations per host generation

When the number of microbial generations per host generation (*T_M_*) was increased to 200, we observed that in all instances the fitness of both host and microbe increased relative to *T_M_* = 1 (Fig. 2). We found that increased microbial generation counts led to a less severe decline in both host and microbial fitness, and lead to the homogenization of the influence of vertical inheritance with regards to host fitness (Fig. 2s–x). In other words, at high values of *T_M_* there was no impact of any level of vertical inheritance on host fitness regardless of environmental conditions (Fig. 2, Supplementary Fig. 8). We found that microbially derived host fitness was typically much higher than when host fitness was entirely derived from the host’s own genetic trait value (Fig. 2s–x). At the same time, microbial fitness remained highest under higher levels of vertical inheritance (Fig. 2m–r). As with the *T_M_* = 1 scenario, we found that when *T_M_* = 200 host fitness was always greater when derived from the microbiome than from the host’s own trait value (Fig. 2s–x).

Similar to where the microbial generation counter per host generation was 1, we found that host fitness was higher under autocorrelated environmental conditions when *T_M_* = 200; however, there was no variation across levels of vertical inheritance, and the fitness improvements were relatively marginal (Supplementary Fig. 7d–f).

### Dynamics of diversity

Over time when the number of microbial generations per host generation (*T_M_*) was 1, the average per-host alpha diversity initially declined at higher levels of vertical inheritance (*X*) followed by a plateau (Fig. 3a–f). Most notably, when there was complete and solely vertical inheritance (*X* = 1), there was often a substantial decline in alpha diversity. Conversely, when *T_M_* = 200 diversity largely remained static across the environmental conditions (Fig. 3g–l). Broadly, low values of vertical inheritance resulted in higher levels of alpha diversity regardless of environmental conditions.

### Influence of microbial generation count on final fitness

**Figure 4.**
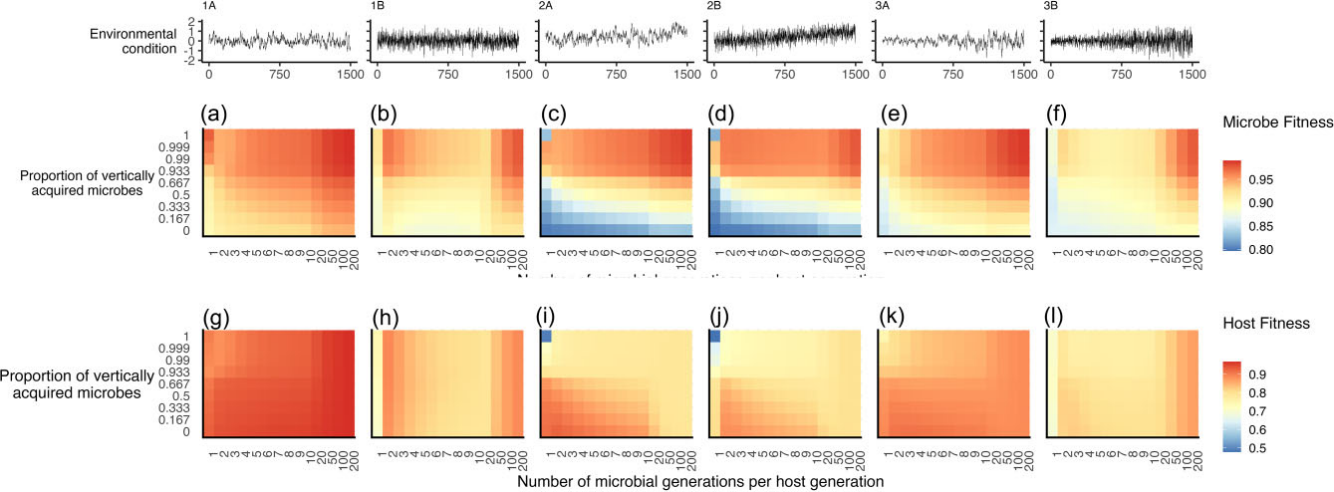
Microbe fitness at host generation 1500 in response to both vertical inheritance proportion and number of microbial generations per host generation (a–f), host fitness at generation 1500 (g–l) in response to both vertical inheritance proportion and number of microbial generations per host generation. Environmental scenarios 1A–3B are as described in Fig. 2.

Generally, we found that higher numbers of microbial generations per host generation (larger *T_M_*) led to higher fitness of both hosts and microbes. Under scenario 2 (increasing mean environment, Fig. 4—panels 2A, 2B) complete vertical inheritance in tandem with *T_M_* = 1 resulted in substantially lower fitness than other scenarios (Fig. 4c, d, i, j) for both host and microbe. In this scenario, and also (albeit to a lesser extent) scenario 3 (increasing variance), low generation counts and low vertical inheritance both lead to lower microbial fitness. Generally, we observed that host fitness was higher when considering highly autocorrelated environmental conditions (Fig. 4g, i, k), whereas randomly generated environmental conditions resulted in lower host fitness (Fig. 4h, j, l).

### Influence of microbial generation count on final diversity

We found that low values of either vertical inheritance (*X*) or microbial generations per host generation (*T_M_*) maximized alpha diversity, while high values of *T_M_* resulted in marginally lower diversity (Supplementary Fig. S9). At the same time, scenarios with both high values of vertical inheritance and low microbial generation counts (Supplementary Fig. S9) tended to have lower diversity than other scenarios. Typically, where *T_M_* = 1, there was greater variation in alpha diversity but it was largely static over time, with the notable exception where *X* = 1 (Supplementary Fig. S10). Similar results were observed where *T_M_* = 200, although vertical inheritance had less influence and variation in diversity was less than where *T_M_* = 1 (Supplementary Fig. S10). We also found that at very high values of *T_M_*, diversity tended to be higher under randomly generated (stochastic) environmental conditions or lower levels of vertical inheritance (Supplementary Fig. S11). Conversely, diversity was higher in autocorrelated environmental conditions where microbial generation count ranged from 1 to 8 and vertical inheritance was high (Supplementary Fig. S11).

## Discussion

Understanding the formation and eco-evolutionary processes of holobionts is a key objective in the study of host-associated microbiomes (Leonard et al. 2024). Using simulations, we incorporate microbial selection into an agent-based model to explore the factors that shape the fitness of both hosts and their associated microbiomes. We assess the impact of increasing the number of microbial generations per host generation, and show that this increases the diversity of microbes within each host and, in turn, facilitates host adaptation to changing environments. We also illustrate that host fitness is higher when environmental change is more predictable (Supplementary Fig. S7). Our work builds upon that of Bruijning et al. (2022), Daybog and Kolodny (2023) and Zeng et al. (2017) by (1) incorporating a large number of microbial generations per host generation and (2) incorporating microbial selection within both the host and the environment (but see Sieber et al. (2019) for an exploration of neutral processes in shaping holobiont structure). Our approach to microbial selection enables microbes to respond differently to selection within the host vs. the environment. We show here that consideration of both direct microbial selection and microbial generation count per host generation is important to understanding how host-microbe systems can evolve under varying environmental conditions.

### Diversity

Our results are somewhat intuitive with regards to microbial alpha diversity and support those of Zeng et al. (2015, 2017). First, under complete vertical inheritance (*X* = 1)—where offspring acquire their microbes completely from their parents and there is no environmental input—and when the number of microbial generations per host generation (*T_M_*) is extremely low, alpha diversity tends to be very low (Fig. 3a–f). This occurs because there are very few opportunities for a host to gain new microbes—thus when a microbe is lost, it is effectively lost permanently, and so diversity can only decline. More generally, high levels of vertical inheritance limit the number of microbial migrants from the environment at birth and result in low levels of alpha diversity. We find that the highest alpha diversity is therefore observed at low levels of vertical inheritance and low numbers of microbial generations per host generation, as environmental colonization of the host is maximized, without subsequent host filtering over multiple microbial generations to reduce the microbial diversity. These results are broadly in line with those observed by Zeng et al. (2015).

In our simulations, the environment experienced by the microbiome is a linear interpolation of the environmental conditions at each host generation. When a community undergoes an instantaneous major environmental change (such as where there is only 1 microbial generation per host generation with a randomly generated environment), this leads to a loss of much of the diversity in the microbiome. Our results align with research into mammalian microbiomes, where post-disturbance alpha diversity is 43% of the pre-disturbance community (Jurburg et al. 2024). In contrast, for microbial communities with multiple generations per host generation, the smooth interpolation between environmental values from one host generation to the next reduces the impact of the disturbance on the microbial community, generally promoting higher microbial diversity relative to fewer microbial generations per host generation. Furthermore, even when disturbances are strong enough to have an impact when there are 200 microbial generations per host generation, the high generation count provides a recovery phase to communities. Once again, these results align with those of mammalian microbiomes, which exhibit recovery of almost all pre-disturbance diversity within ∼50 days post-disturbance (Jurburg et al. 2024).

We note that there is also typically higher microbial diversity in the autocorrelated environmental conditions relative to the randomly generated counterpart under lower microbial generation counts. This may be explained by the Intermediate Stochasticity Hypothesis (Santillan and Wuertz 2022), which posits that intermediate levels of environmental variability can maximize diversity through temporal niche differentiation—where under relatively static environments one or few specialists can dominate (this occurs under many microbial generations in an autocorrelated environment), and under highly stochastic environments nothing or few things can survive. In relation to our environmental conditions, a randomly (non-autocorrelated) environmental condition represents a highly stochastic environment (especially where there are few microbial generations per host generation), while a static environment is an entirely predictable/non-stochastic environment. An autocorrelated environment is somewhat intermediate to these two extremes—whereby the average magnitude in environmental change between two successive environmental conditions will be relatively low where microbial generation counts are relatively low. However, where there are many microbial generations per host generation, the average magnitude of change is extremely low—resulting in a relatively static environment where diversity can decline. Therefore, high vertical inheritance in tandem with low microbial generation counts should increase diversity in an autocorrelated environment relative to the non-autocorrelated environment (Supplementary Fig. S11)—as these scenarios present both continuity within a microbial community and an intermediate stochastic environment. Conversely, high microbial generation counts in these autocorrelated environments should have lower diversity than the non-autocorrelated counterpart due to the very low change in environmental conditions between microbial generations, resulting in more specialist microbes dominating the community (Supplementary Fig. S11). This means that, on average, the environmental changes are more predictable in an autocorrelated than a random environment. Similar results have also been observed in macroalgal microbiomes, where environmental predictability (as measured through the degree of temporal autocorrelation in temperature) is a driver of holobiont diversity (Pearman et al. 2024).

### Fitness

We demonstrate that host fitness derived from the microbiome was almost always greater than fitness derived from a host’s own trait value. Although expected, this result demonstrates that the microbiome can be important in buffering hosts to environmental change. We found both host and microbe fitness increased with higher numbers of microbial generations per host generation (i.e. *T_M_*), such a result makes sense because high values of *T_M_* ‘spread’ the environmental change over more microbial generations, as described above. Such ‘spread’ allows for gradual adjustment and changes in the microbiome, whereas when environments undergo sudden drastic changes, this leads to a stronger disturbance effect and thus greater initial changes in the microbiome, leading to the loss of retained microbes. We hypothesize that the gradual adjustment of the microbiome with a higher number of microbial generations can lead to cross-generational priority effects which, in our simulations, may provide a benefit to hosts if those microbes subsequently become beneficial when environmental conditions change. Priority effects occur when the order and timing in which species arrive into a community shapes the resulting community; e.g.—early colonizers of a microbe-free host may have a competitive advantage due to being able to establish without competing strongly for niche space (Fukami 2015).

For example, take a scenario where the environmental conditions transition from 0.2 to 0 and back to 0.2 over two host generations—if vertical inheritance is sufficiently high, some of those microbes suited to an environment of 0.2 may persist throughout the environmental change by virtue of starting in high abundance (thus negative selection may reduce, but not fully remove, these microbes in the host), resulting in the hosts deriving a benefit from the priority effects when the environment returns to 0.2. This possibility is further amplified by having very large microbial communities, resulting in a greater number of ‘persister’ microbes which have the advantage of not needing to recolonize the host. These cross-generational priority effects appear to be stronger in our autocorrelated environments, which tend to have both higher host and microbe fitness (Fig. 4)—this is likely because autocorrelation increases the ‘continuity’ of environmental conditions and leads to a weaker disturbance effect as a result of less sharp changes in selective pressures imposed by the environment. This is perhaps also why we observe higher fitness variance under autocorrelated environmental conditions when the number of microbial generations is low (Supplementary Fig. S12, Supplementary Fig. S13), as these cross-generational priority effects enable maintenance of higher levels of diversity upon which selection can act. Similar processes have been observed in microbial communities associated with floral nectar, where priority effects have been observed to persist across multiple floral generations (Toju et al. 2018). Our results are also in line with those observed by Bruijning et al. (2022)—their Fig. 6—who found higher phenotypic variance in lower vertical transmission/high microbial generation count scenarios—although notably our results are dependent on the specific environmental conditions being studied, with randomly generated environments being most aligned with Bruijning et al. (2022) (Supplementary Fig. S12, Supplementary Fig. S13).

Interestingly, broadly across our simulations, we found that under scenarios where there was only one microbial generation per host generation, there was a conflict between the influence of vertical inheritance on host and microbe fitness, where high vertical inheritance maximized microbial fitness within the host but tended to have the opposite effect on host fitness. This result highlights the contrast, and potential conflicts, that can arise between hosts and their microbes. Importantly, because we model host fitness as derived from the relationship between the average trait value of a host’s microbial community and its distance to the environmental condition, it is best for a host to completely replace its microbiome when the microbial generation count is low so that it can acquire a microbiome that is more environmentally fit. Notably, this does not provide benefits for the microbiome, which is suited to the environment more than the host, but allows the host to maximize the gains from the microbiome. In contrast, under low microbial generation counts microbial fitness tends to be highest under high vertical transmission (with the exception, again, being where vertical inheritance is complete and there is only a single microbial generation per host generation) because there is continuity in the microbial communities across host generations, allowing for microbial selection to operate across multiple host generations and therefore allowing for host microbiomes to adapt to the host.

We note that it is possible that the host and microbe fitness gains we see from increasing microbial generation counts (*T_M_*) could be primarily driven by changes in effective vertical inheritance. Effective vertical inheritance is the term we use to describe the proportion of parentally acquired microbes remaining in a host microbiome at the point at which that host reproduces. Such a value is expected to decline over time due to the loss of incumbent microbes and replacement with microbes from the environment (see microbiome heritability; Bruijning et al. 2022). Thus, even when a host acquires their initial microbiome entirely from their parents, if hosts gain microbes from the environment over their lifetime after 100 generations only ∼10% of the parentally acquired microbes may have any direct descendants within the final host microbiome. To test the argument that effective vertical inheritance drives holobiont fitness rather than microbial generation count, we simulated equivalent effective vertical inheritance scenarios, but one where *T_M_* = 1 and the other where *T_M_* = 200 (Supp. Information; Supplementary Fig. S14). Our results demonstrate that there is usually higher fitness in both host and microbe in the latter, supporting our hypothesis that the number of microbial generations per host generation acts as a buffering effect to environmental change.

### Outlook and next steps

By extending existing models of microbiome assemblages, we have made significant contributions to our understanding of how microbial generation count and changing environments impact both host and microbe fitness in simulated holobionts. Our current model does not enable variation in the strength of selection between the host and environment and we encourage future exploration of this factor, as different selection strengths of host/environment upon the microbiome may have significant consequences for holobiont formation and evolution. For example, in order to avoid negative/parasitic microbes, it might be important that hosts have a stronger selective influence upon the microbiome than does the environment. Alternatively, microbes which are subject to strong environmental selection may benefit hosts more by providing a stronger buffering influence.

In addition, although we consider the degree of autocorrelation and variance of environmental conditions in shaping fitness, a systematic exploration of these factors is warranted in future studies in order to better understand the relationship between the time scale of variation relative to host/microbe generation times in shaping the selective landscape for holobionts.

Our model, which builds off dynamics of Wright-Fisher population models, is restricted to the limitations of this framework (specifically non-overlapping generations and fixed population sizes), and there is clear need to further extend this and other existing models in order to understand how microbiome assemblages can evolve, and may buffer hosts from the impacts of a changing environment. Most notably, our model, as presented here, is fundamentally an ecological model and does not structure either microbial or host traits as genetic traits—thus we suggest that this is one of the most pressing elements to focus on in the future. For example, many studies to date, including this one, have made the key assumption that a host's fitness is entirely derived from its microbiome. This is a necessary simplification, because it sidesteps the need to develop a quantitative genetic model (inclusive of mutation) to describe host evolution within a holobiont system. Once developed, and as previously suggested by Daybog and Kolodny (2023), an exciting further extension of such a host genetic model would be to allow the fitness contributions of the microbiome to the host to vary across hosts and evolve over time, to test the conditions under which host-microbiome partnerships, i.e. holobionts, are most likely to evolve. In addition, future work enabling microbial evolution would be worthwhile—as alongside host genetics, it would delve directly to the heart of questions of co-evolution and holobiont evolution.

## Supplementary Material

fiaf089_Supplemental_File

## Data Availability

Code for reproducing our analyses and simulations, and example code to demonstrate the model is available at: https://wpearman1996.github.io/Holobiont_Bookdown/. Archived code is available at: https://zenodo.org/records/17102729.
