# Supplementary material for: Within-host microbial selection and multiple microbial generations buffer the loss of host fitness under environmental change
Source: FEMS Microbiol Ecol. 2025 Sep 8;101(10):fiaf089. doi: 10.1093/femsec/fiaf089 (PMC12451448; doi:10.1093/femsec/fiaf089)
Supplement: fiaf089_Supplemental_File [file fiaf089_supplemental_file.docx]

**Supplementary Information**

**Generation of environmental conditions**

We tested the impact of six different environmental scenarios on host and microbe fitness and microbial diversity. These scenarios were (1) ‘unchanging’ environments, where the environment fluctuates around a mean, creating variation around that mean, (2) an increasing mean environment where the mean environmental condition increases over time – with variation around this changing mean, and finally (3) an environment where the variance around the mean increases over time but the mean itself does not. For each of these scenarios we have two versions, (A) a high autocorrelation version where the environmental conditions from each point to the subsequent are highly autocorrelated or (B) a ‘random’ variation version where the environmental condition is drawn independently of the previous time point. For the random, no autocorrelation environment, the burn in drew environmental values at random from a normal distribution with a mean of 0 and standard deviation of 0.5. Environmental conditions from generations 201-1,500 were then generated as follows: for the first random, no autocorrelation environment (Fig. 2 - panels 1B, 2B, 3B), values continued to be drawn from a normal distribution with a mean of 0 and standard deviation of 0.5 (Fig. 2 - panel 1B), for the second, an increasing mean from 0 to 1 and standard deviation of 0.5 (Fig. 2 - panel 2B), and for the third, a mean of 0 and increasing standard deviation from 0.3 to 0.84 over the simulation (Fig. 2 - panel 3B). In the case of the high autocorrelation environment, burn in values were drawn using a bespoke autoregressive integrated moving average (ARIMA) function to allow for varying standard deviations with an AR component of 0.9, MA component of 0, and with standard deviation of 0.2 and a mean of 0. Following burn in, for the first autocorrelated environment, values continued to be drawn with a standard deviation of 0.2 and a mean of 0 (for a no-net change environment, Fig. 2 - panel 1A), for the second, a varying mean from 0 to 1.0 (increasing mean environment, Fig. 2 - panel 2A), and for the third, a mean of 0 and a standard deviation increasing from 0.1 to 0.3 (for the increasing variation condition, Fig. 2 - panel 3A). These values were chosen to match the overall standard deviations across the low and high autocorrelation environmental conditions.

**
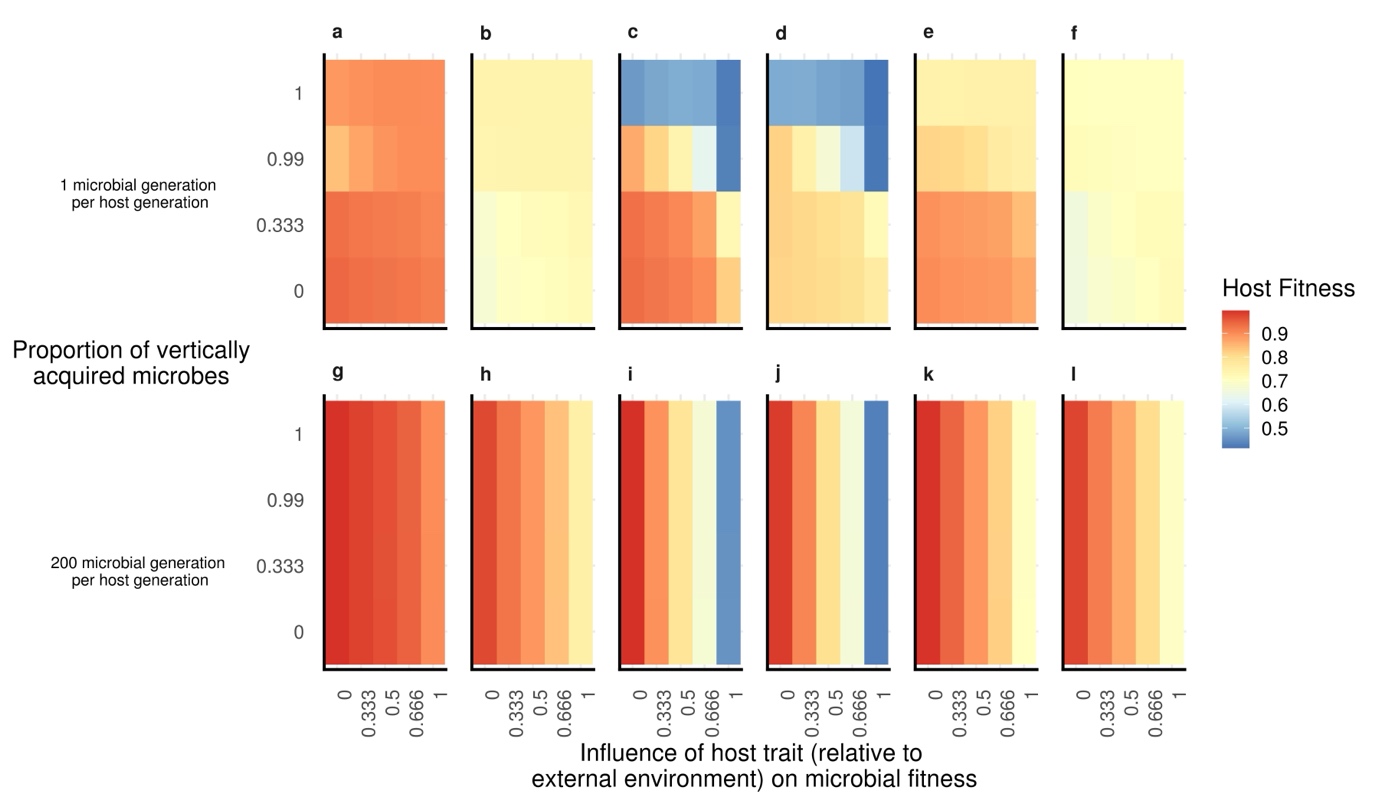
**

Supp. Fig. 1. Host fitness at host generation 1,500 in response to both vertical inheritance proportion (*X*) and the relative influence of the host trait value (*I,* note that where *I* = 0 the environmental is entirely responsible for microbial fitness) on microbial fitness at 1 microbial generation per host generation (a-f) and 200 microbial generations per host generation (g-l). Host fitness decreases with the influence of the external environment on the microbiome. Notably, vertical inheritance plays a greater role where *T_M_ =*1 because the environment is less able to contribute to the microbiome at high values of *X*. When these scenarios are combined with high values of *I* the fitness loss is more notable due to an increased disparity between microbiome composition and microbial fitness. This is because the host microbiome is unable to respond to changes in the environment both through direct selection (i.e., through values of *I* < 1 enabling environmental selection), and through indirect selection (i.e., vertical inheritance ≪1 enabling acquisition of microbes from the environment). This is clearest in c/d, and i/j – as these scenarios are where the environmental condition deviates from a mean of zero over time – resulting in environmental microbes responding to environmental selection, but not host microbes. Results in the main text relate to an intermediate value of *I* = 0.5. Specific facets relate to different environmental conditions, see Fig. 2 in main text.

**
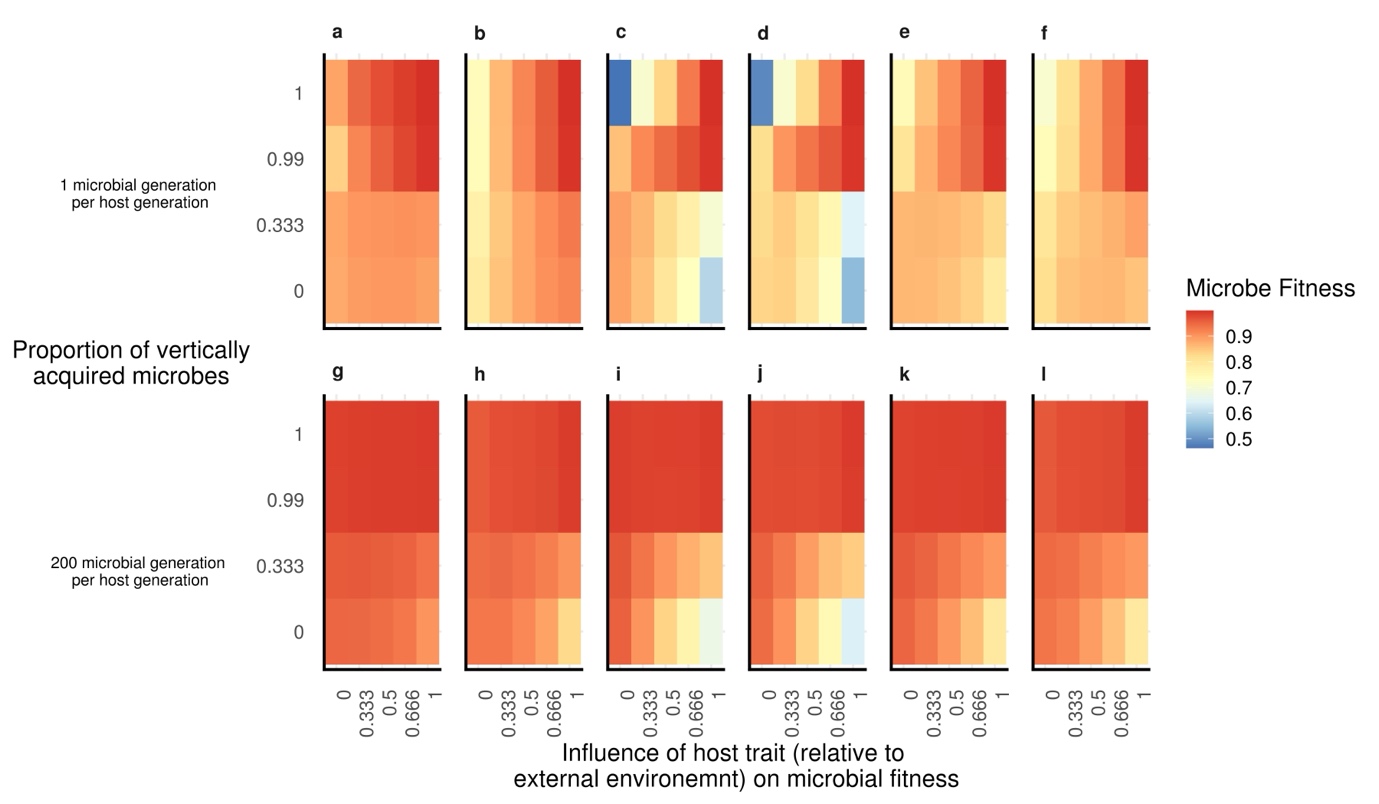
**

Supp. Fig. 2. Microbial fitness at host generation 1,500 in response to both vertical inheritance proportion (*X*) and the relative influence of the external environment (*I*) on microbial fitness at 1 microbial generation per host generation (a-f) and 200 microbial generations per host generation (g-l). Microbe fitness is highest under high vertical inheritance (*X*) and high values of *I* as the external environment has no influence and microbes are largely parentally inherited – resulting in selection for the microbes best suited to the host genetic trait value (which is static over time, unlike the environment). For example, where *X* = 0 and *I* = 1 host-associated microbes are acquired entirely from the environment (which has no influence on microbe fitness within the host), resulting in complete mismatch between the source of microbes and the source of selection. Results in the main text relate to an intermediate value of *I* = 0.5.


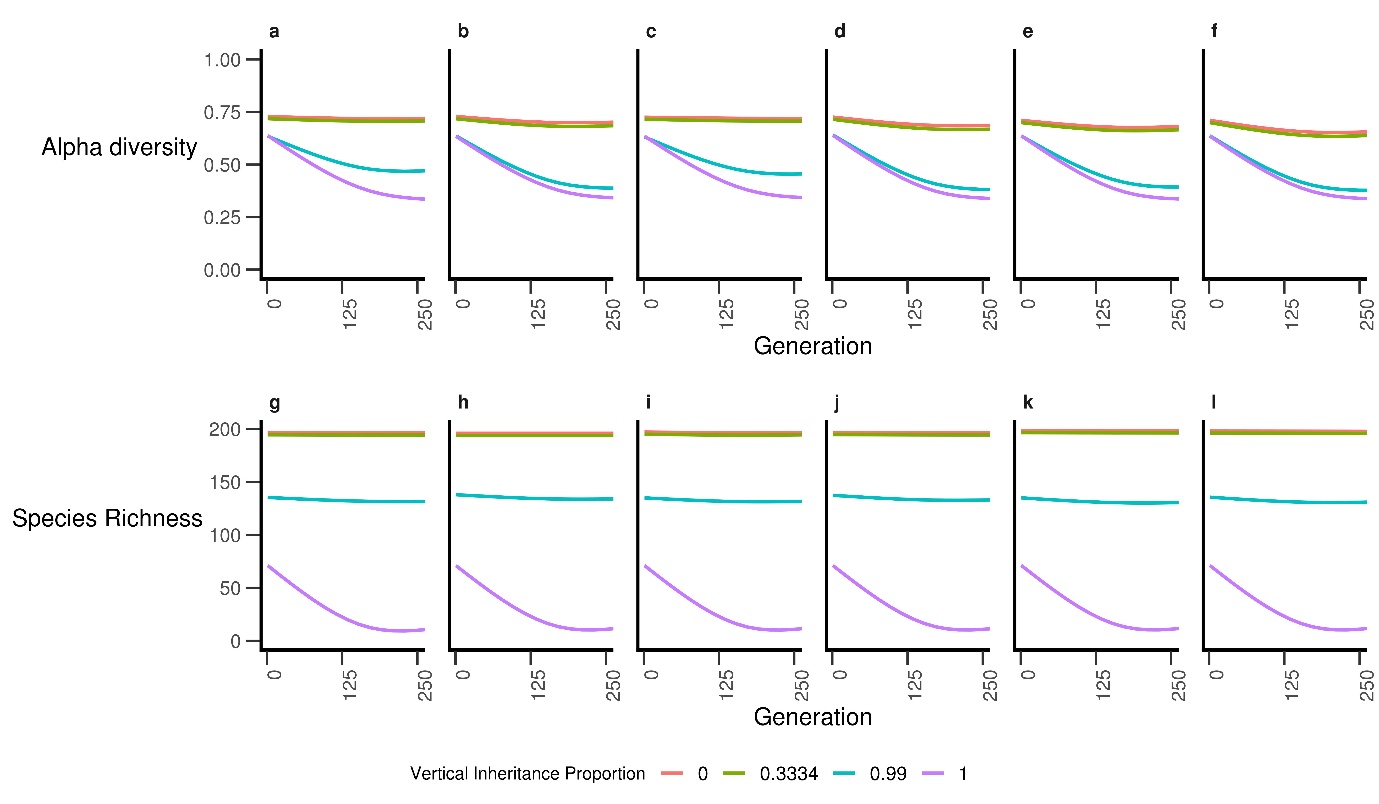


Supp. Fig. 3 Alpha diversity as either scaled Shannon-Weiner index (a-f) or species richness (g-l) across a range of values of vertical inheritance over the burn-in period (200 generations), where the number of microbial generations per host generation is 1. Lines represent GAM smoothing, data represents mean values from 20 replicate simulations. Line colour indicates degree of vertical inheritance (*X*). Facets a and b correspond to an unchanging environment with either high autocorrelation between sample points (a) or randomly generated sample points (b), the same order applies with the remaining plots except c and d are increasing mean environments, and e and f are increasing variance environments. This order is identical for g-l.

**
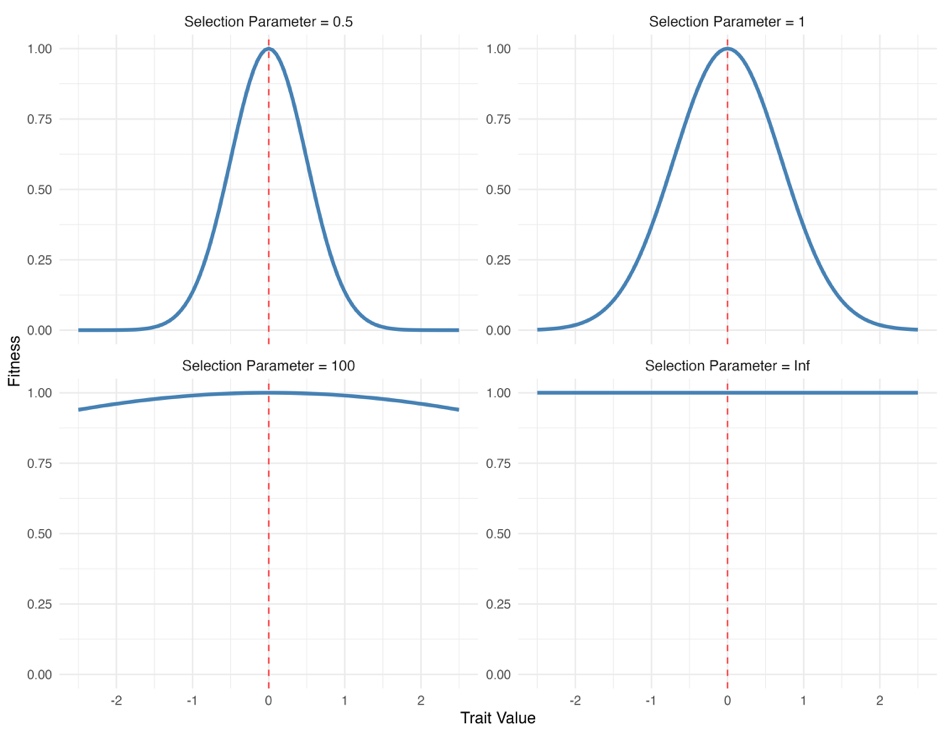
**

Supp. Fig. 4. Fitness landscapes across a variety of strengths of selection, where the optimal trait value is 0 and the possible phenotypes range from -2.5 to 2.5. Note that lower values of the selection parameter result in stronger selection. Results in the main text relate to a medium-strong selection value of *s* = 1.


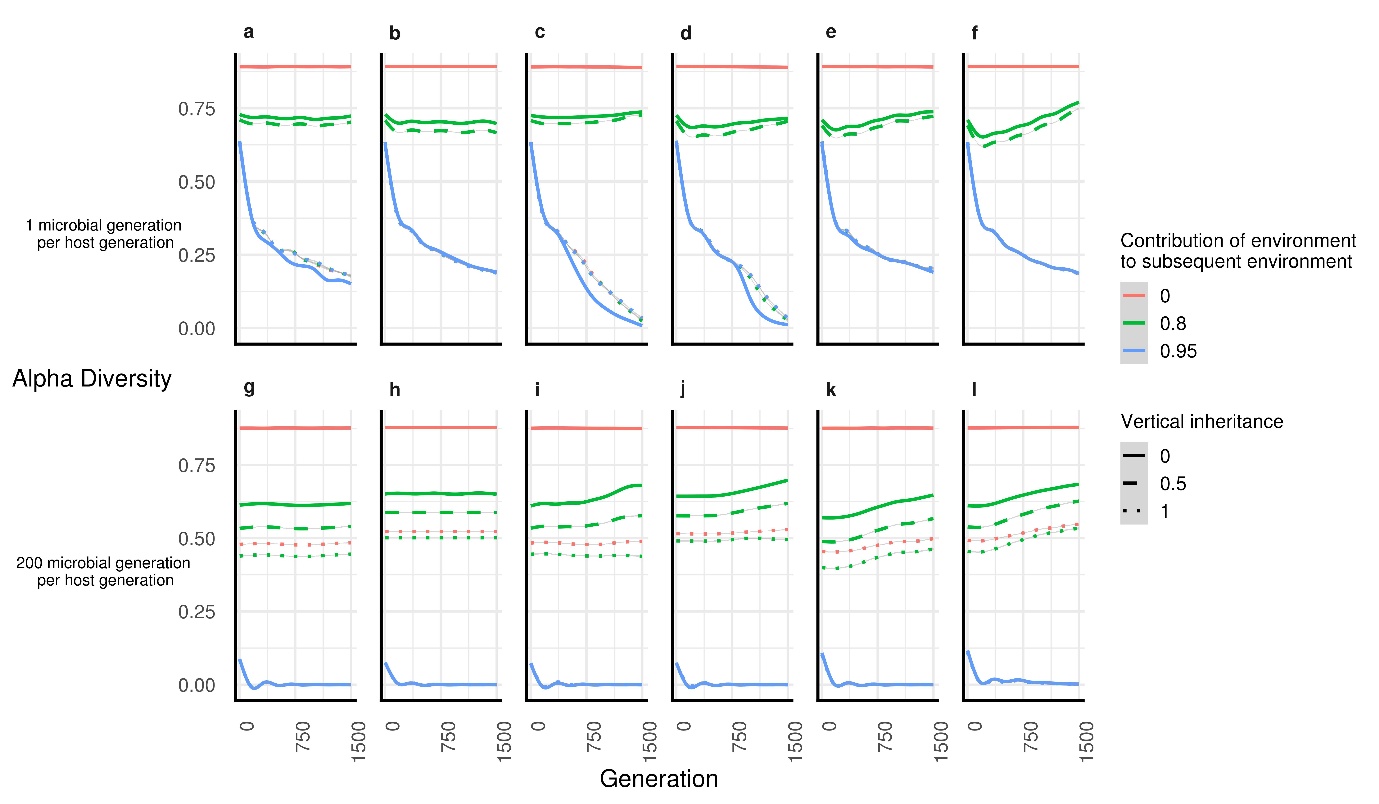


Supp. Fig. 5. Alpha diversity across a range of values of vertical inheritance over the course of the simulations when the number of microbial generations per host generation is 1 (a-f) or 200 (g-l), for a range of values of the contribution of the environment at each time point to the subsequent generation’s environment (*Z*). In all cases, the contribution of host shedding to the subsequent generation’s environment is fixed at 0.05, so a decrease in *Z* means a corresponding increase in the contribution of the fixed environment to the next generation. Lines represent GAM smoothing, data represents mean values from 20 replicate simulations. Line type indicates degree of vertical inheritance (*X*), while colour indicates the relative contribution to the environment in the subsequent generation. Facets a and b correspond to an unchanging environment with either high autocorrelation between sample points (a) or randomly generated sample points (b), the same order applies with the remaining plots except c and d are increasing mean environments, and e and f are increasing variance environments. This order is identical for g-l. In general, alpha diversity is highest when the contribution from the fixed environment is maximized and there is no contribution from the current environment, which has been acted upon by selection and hence is less diverse than the fixed pool. Alpha diversity is correspondingly lowest when there is no contribution from the fixed pool (*Z* = 0.95). Results in the main text relate to an intermediate value of *Z* = 0.8.

**
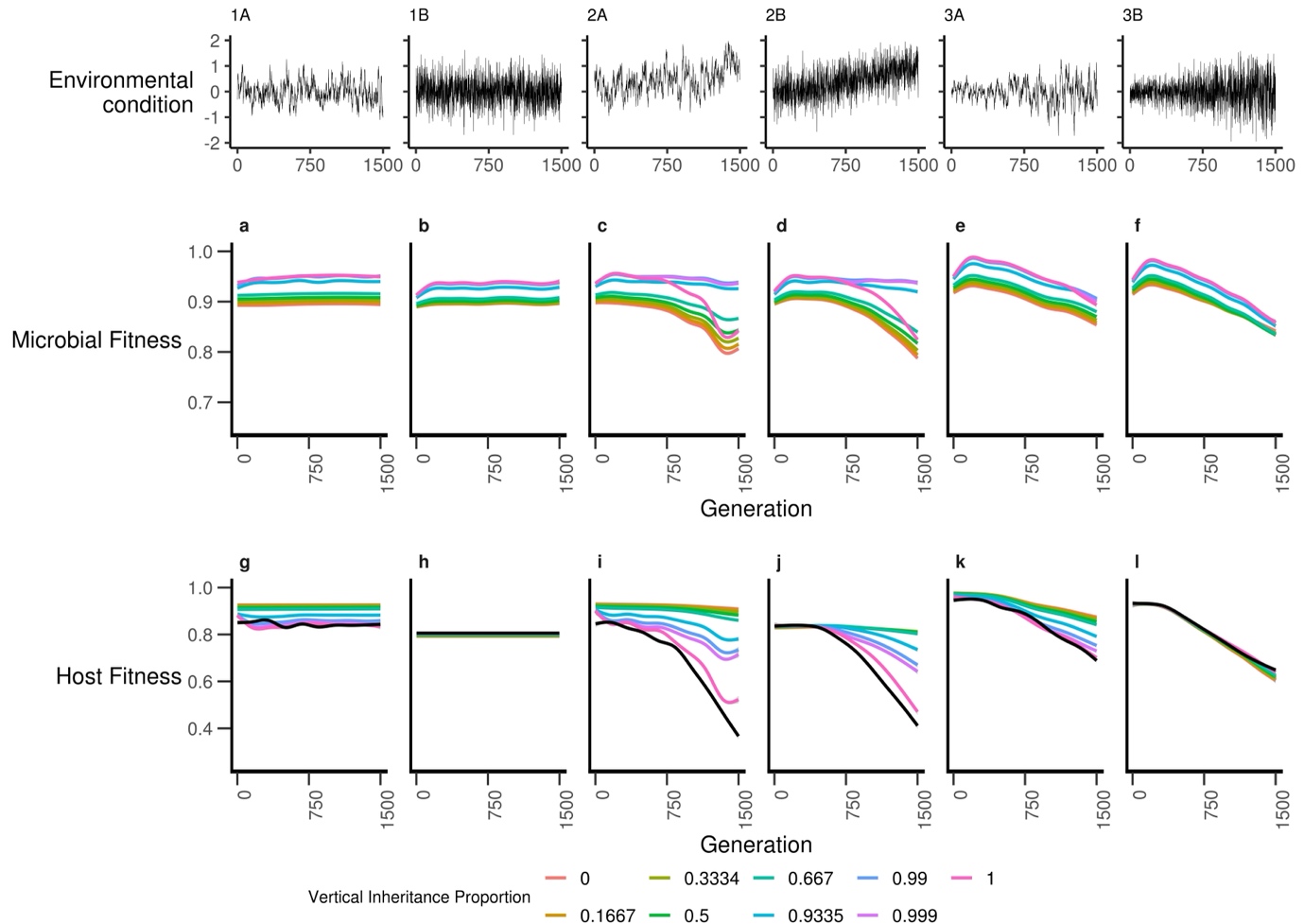
**

Supp. Fig. 6: Microbial fitness (a-f) and host fitness (g-l) over the course of the simulations when the number of microbial generations per host generation is 1. Lines represent GAM smoothing; data represents mean values from 20 replicate simulations where G = 1. Black points in panels g-k are the host fitness where the contribution of the microbiome to host fitness is zero (i.e., G = 0), this value does not affect microbial fitness and as such is not displayed on a-f. The figure is similar to Fig. 2 in the main text, but visualizes a greater diversity of vertical inheritance proportions.

*
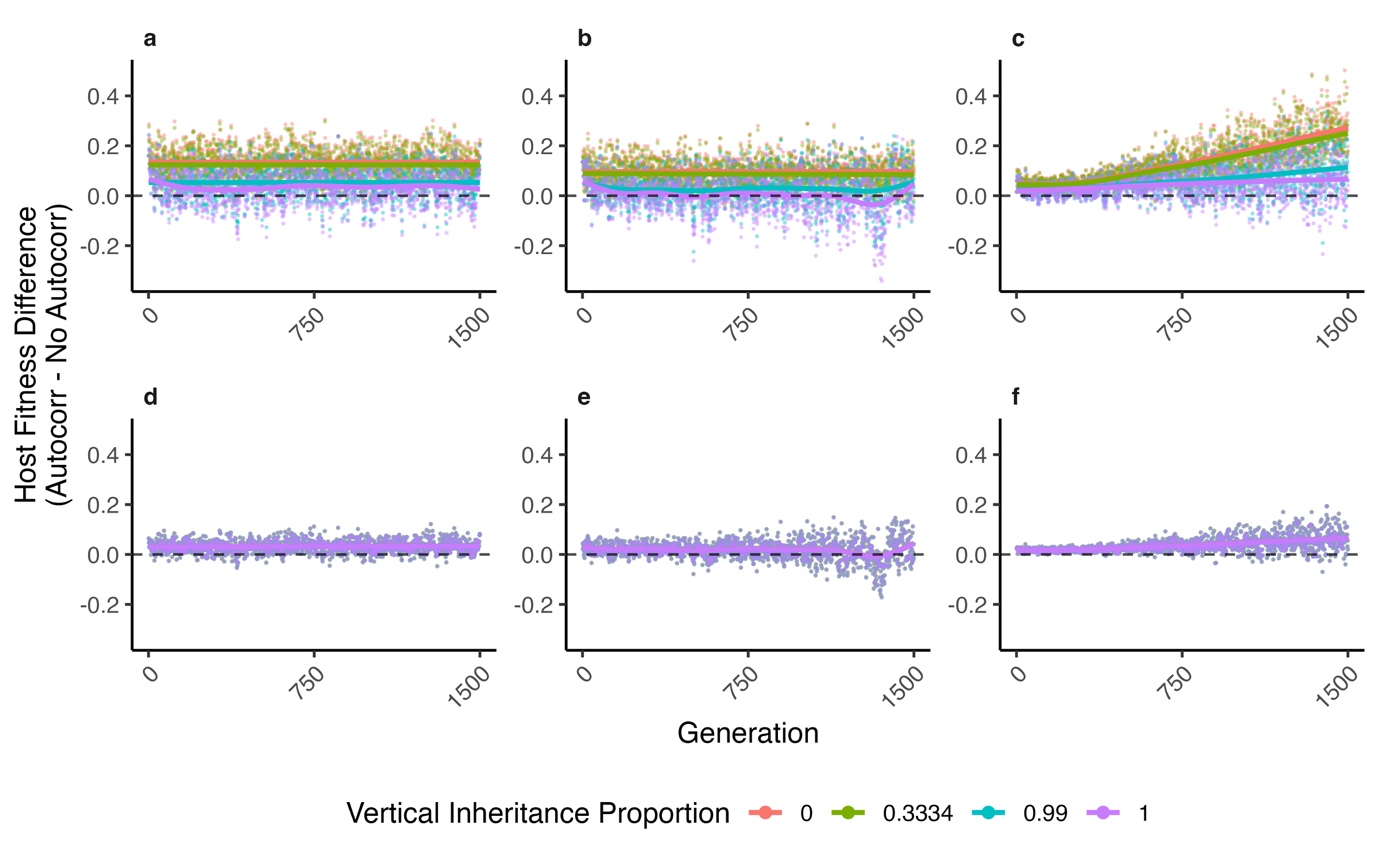
*

Supp. Fig. 7: Change in host fitness between autocorrelated and non-autocorrelated environmental conditions in response to vertical inheritance (*X*). Positive values indicate the degree to which host fitness is higher in an autocorrelated environment, while negative indicate higher host fitness in the stochastic environments. a-c corresponds to scenarios with 1 microbial generation per host generation, and d-f to scenarios with 200 microbial generations per host generation. (a,d) correspond to a no-net change environment, (b,e) to a increasing mean environmental condition, and (c,f) to an increasing variance environment.

**
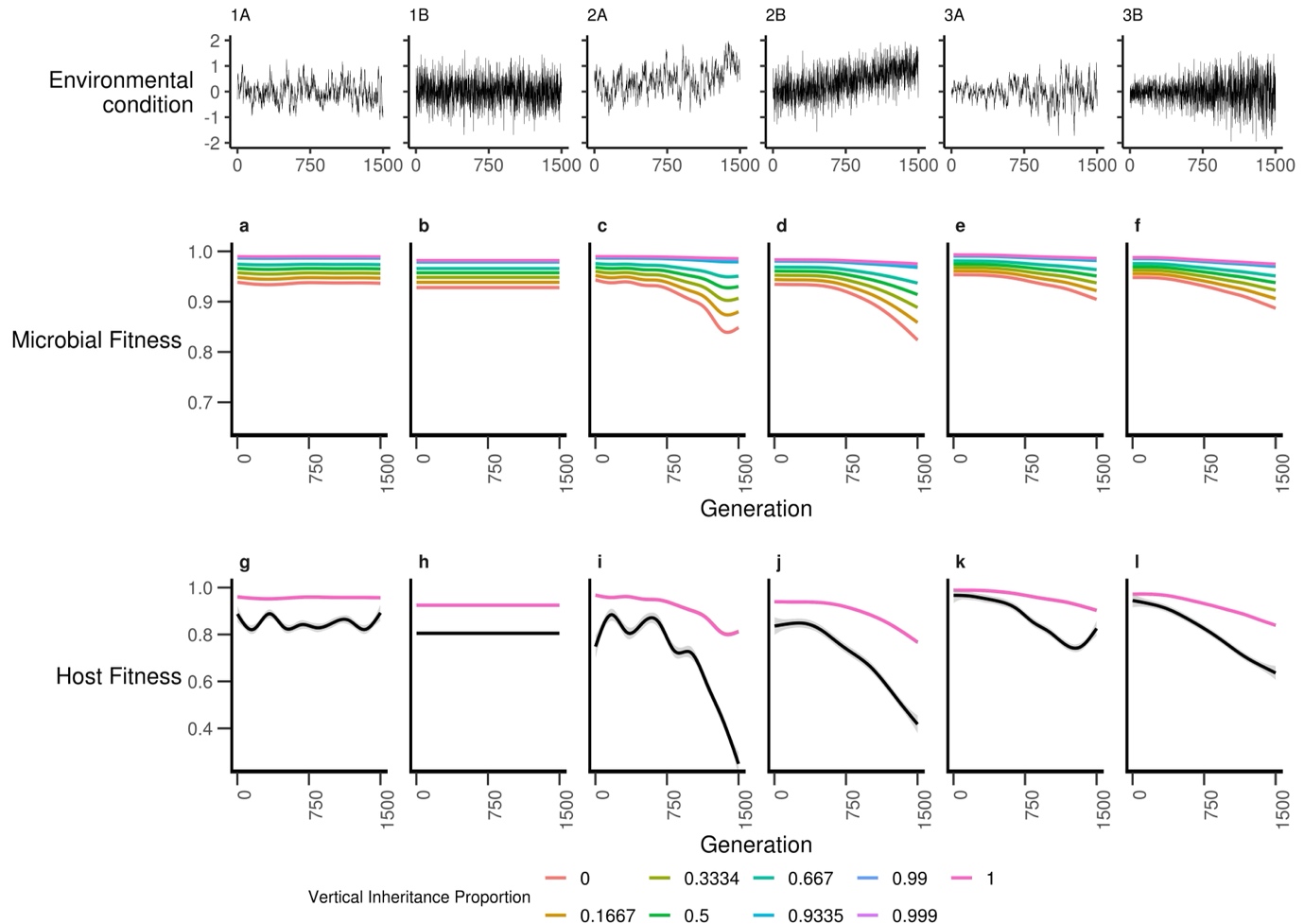
**

Supp. Fig. 8: Microbial fitness (a-f) and host fitness (g-l) over the course of the simulations when the number of microbial generations per host generation is 200. Lines represent GAM smoothing; data represents mean values from 20 replicate simulations where G = 1. Black points in panels g-k are the host fitness where the contribution of the microbiome to host fitness is zero (i.e., G = 0), this value does not affect microbial fitness and as such is not displayed on a-f. The figure is similar to Fig. 3 in the main text, but visualizes a greater diversity of vertical inheritance proportions.

**
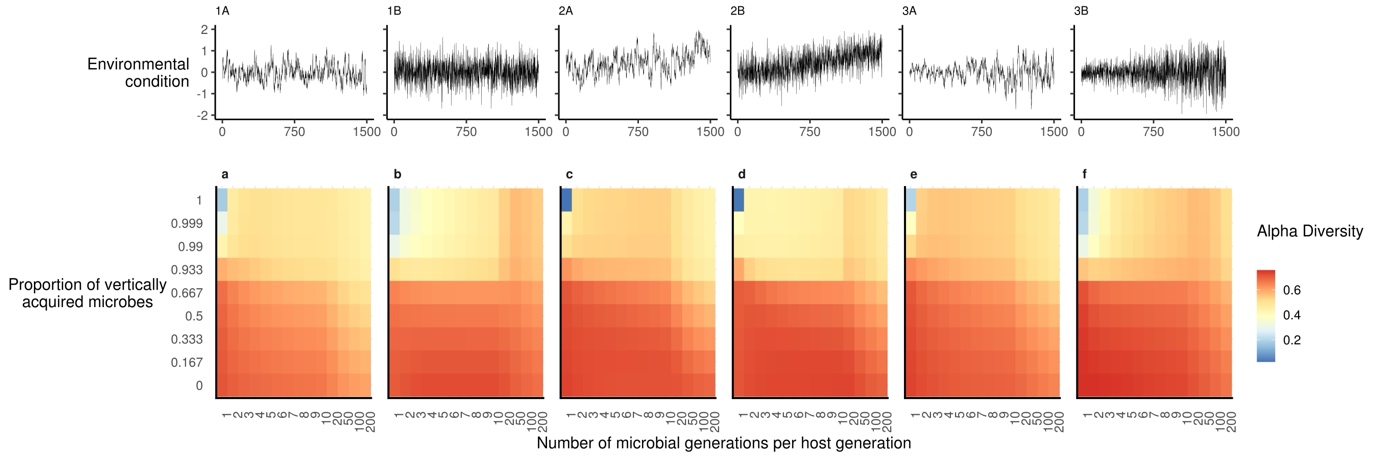
** Supp. Fig. 9. Alpha diversity of microbiomes at host generation 1,500 in response to both vertical inheritance proportion (*X*) and number of microbial generations per host generation (*T_M_*) (a-f).

**
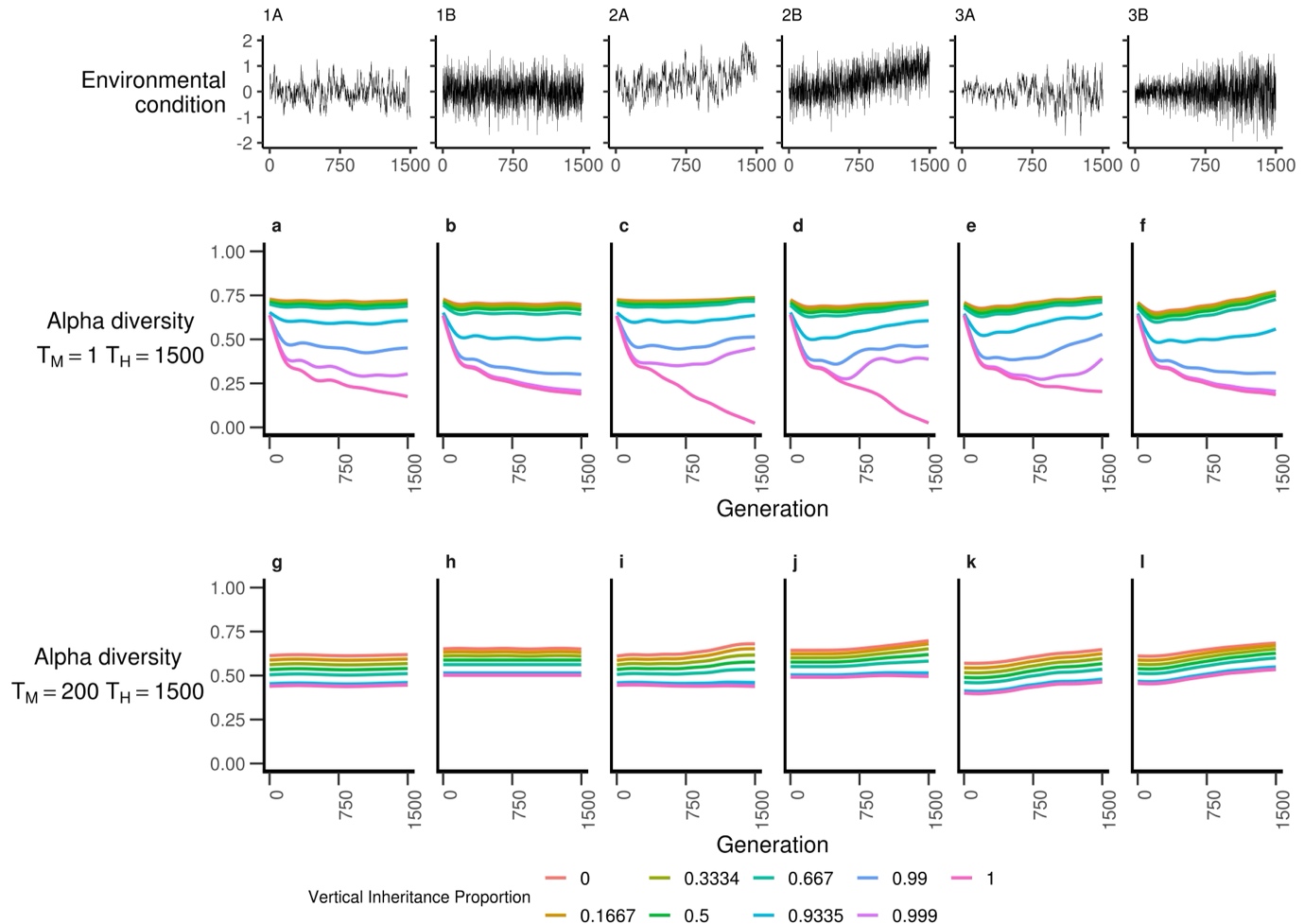
**

Supp. Fig. 10. Alpha diversity across a range of values of vertical inheritance (*X*) over the course of the simulations when the number of microbial generations per host generation is 1 (a-f) or 200 (g-l). Lines represent GAM smoothing, data represents mean values from 20 replicate simulations. Facets a and b correspond to an unchanging environment with either high autocorrelation between sample points (a) or randomly generated sample points (b), the same order applies with the remaining plots except c and d are increasing mean environments, and e and f are increasing variance environments. This order is identical for g-l.


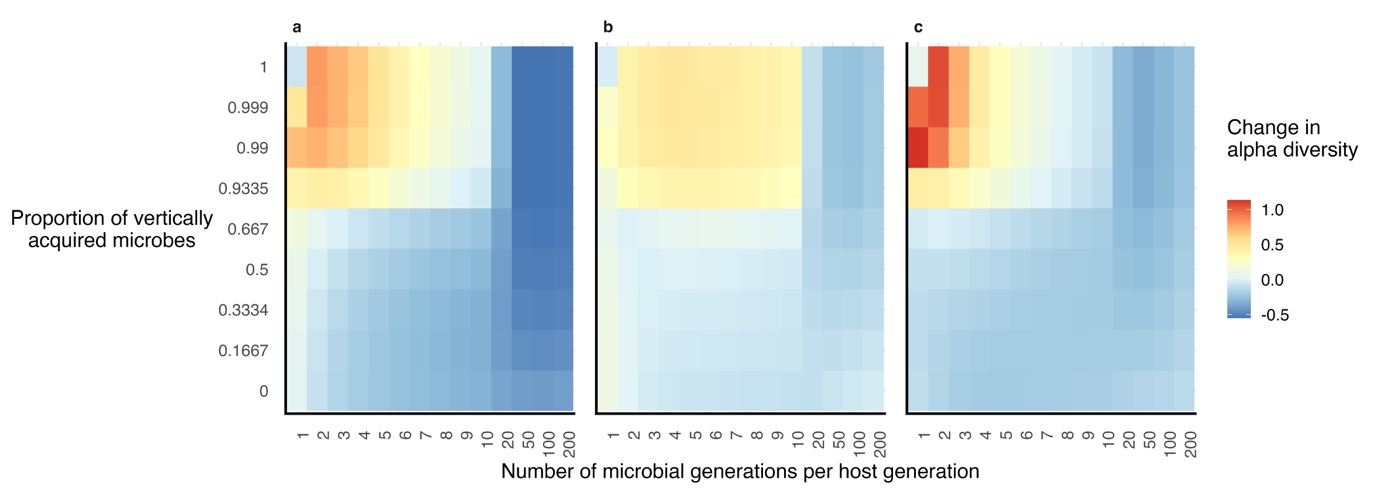


Supp. Fig. 11. Change in alpha diversity at generation 1,500 in response to both vertical inheritance (*X*) and microbial generation count (*T_M_*). Positive values indicate the degree to which diversity is higher in an autocorrelated environment, while negative indicate higher diversity in the stochastic environments. (a) corresponds to a no-net change environment, (b) to an increasing mean environmental condition, and (c) to an increasing variance environment.


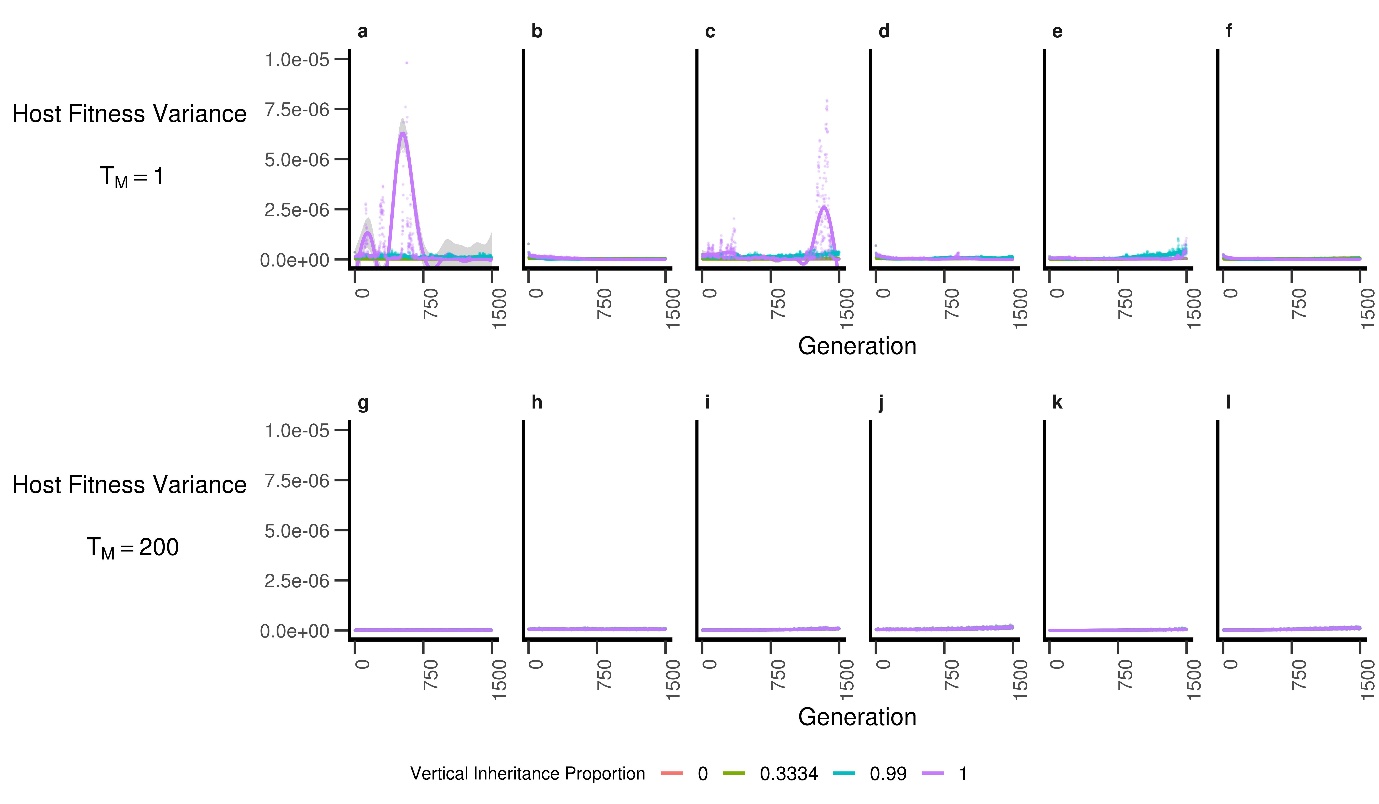


Supp. Fig. 12. Fitness variance within host populations over the course of the simulations where the number of microbial generations per host generation is 1 (a-f) or 200 (g-l). Lines represent GAM smoothing, data represents mean values from 20 replicate simulations. Line colour indicates degree of vertical inheritance (*X*). Facets a and b correspond to an unchanging environment with either high autocorrelation between sample points (a) or randomly generated sample points (b), the same order applies with the remaining plots except c and d are increasing mean environments, and e and f are increasing variance environments. This order is identical for g-l.


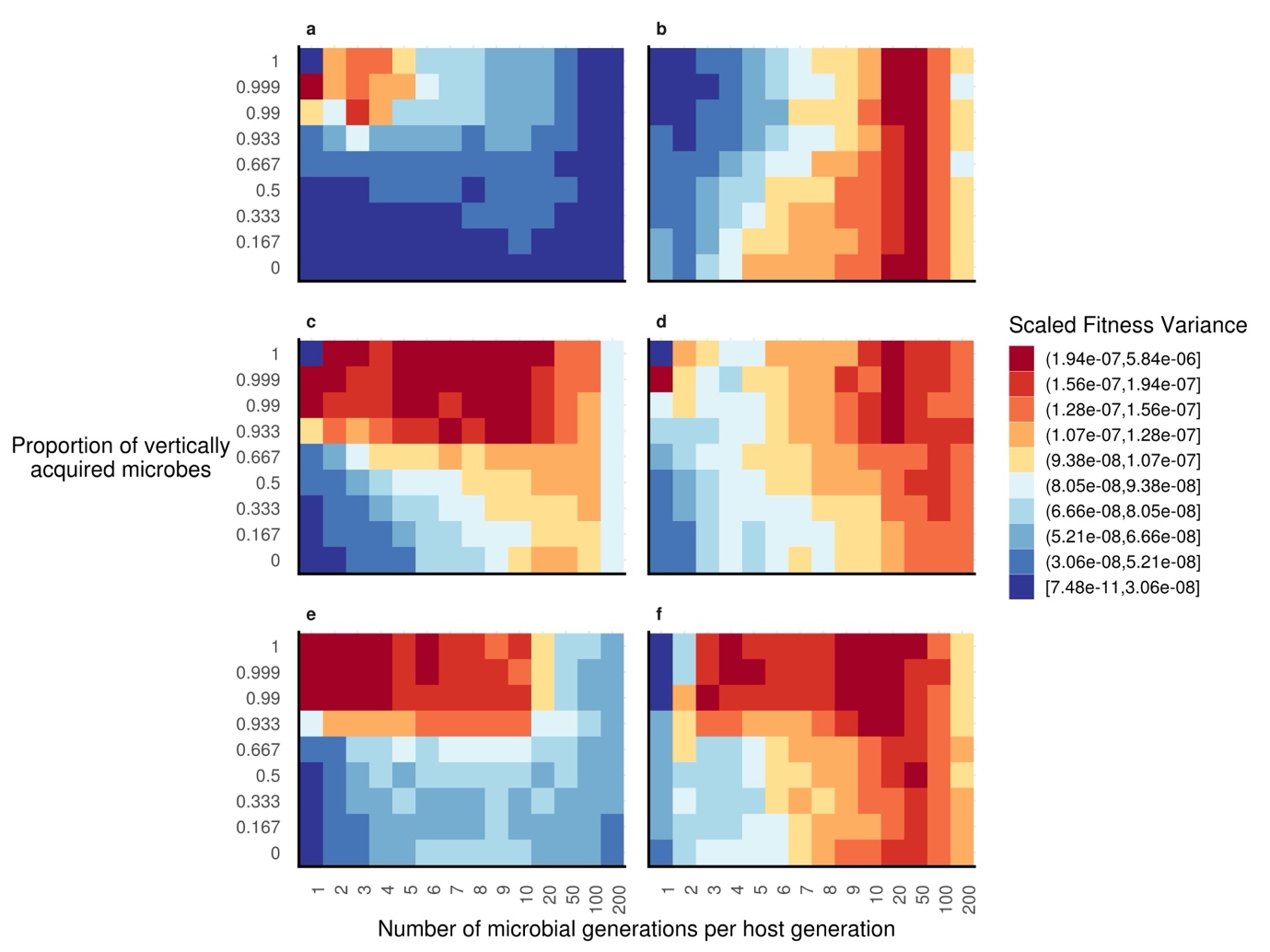


Supp. Fig. 13. Host fitness variance at host generation 1500 for a range of values of vertical inheritance (*X*) and microbial generations per host generation (*T_M_*). Note that because the variance is very low, the values have been binned into equally sized bins, and thus results should be cautiously interpreted. Facets a and b correspond to an unchanging environment with either high autocorrelation between sample points (a) or randomly generated sample points (b), the same order applies with the remaining plots except c and d are increasing mean environments, and e and f are increasing variance environments.

**The impact of effective vertical inheritance on host and microbe fitness**

When a host reproduces, microbes are inherited at proportion *X* from parent to offspring (vertical inheritance), but subsequently at proportion *P* from one microbial generation within the host to the next. We generally expect that *X* and *P* will differ, because colonization of a new host is likely to differ from the microbial turnover in an existing host microbial community. Further, the impact of the initial colonization of a new host will become diluted as the number of microbial generations within the host increases. We therefore tracked the effective vertical inheritance from one host generation to the next as the proportion of the original colonization community that was remaining at the end of the host generation. Vertically acquired microbes were classified as any microbe originating from the host’s direct parent, thus if a parent sheds a microbe into the environment – which then persists for a few microbial generations before colonizing the offspring – this would also be classified as vertical inheritance. We numerically (*cf.* analytically) quantified effective vertical inheritance independently for each value of *X* and each environmental scenario at the 500^th^ host generation by labelling parentally acquired microbes and tracking their abundance in each scenario across a host generation. Following this we ran an additional set of simulations for one microbial generation per host generation (*T_M_* = 1), where *X* was set as the effective vertical inheritance equivalent as quantified for *T_M_* = 200, and compared host and microbe fitness to their fitness at *T_M_* = 200.

Overall, both host and microbe fitness increase with increasing numbers of microbial generations, in agreement with our previous conclusions (Supp. Fig. 11). These results suggest that high values of *T_M_* result in fitness gains regardless of the level of effective vertical inheritance, supporting our hypothesis that the number of microbial generations per host generation acts as a buffering effect to environmental change.


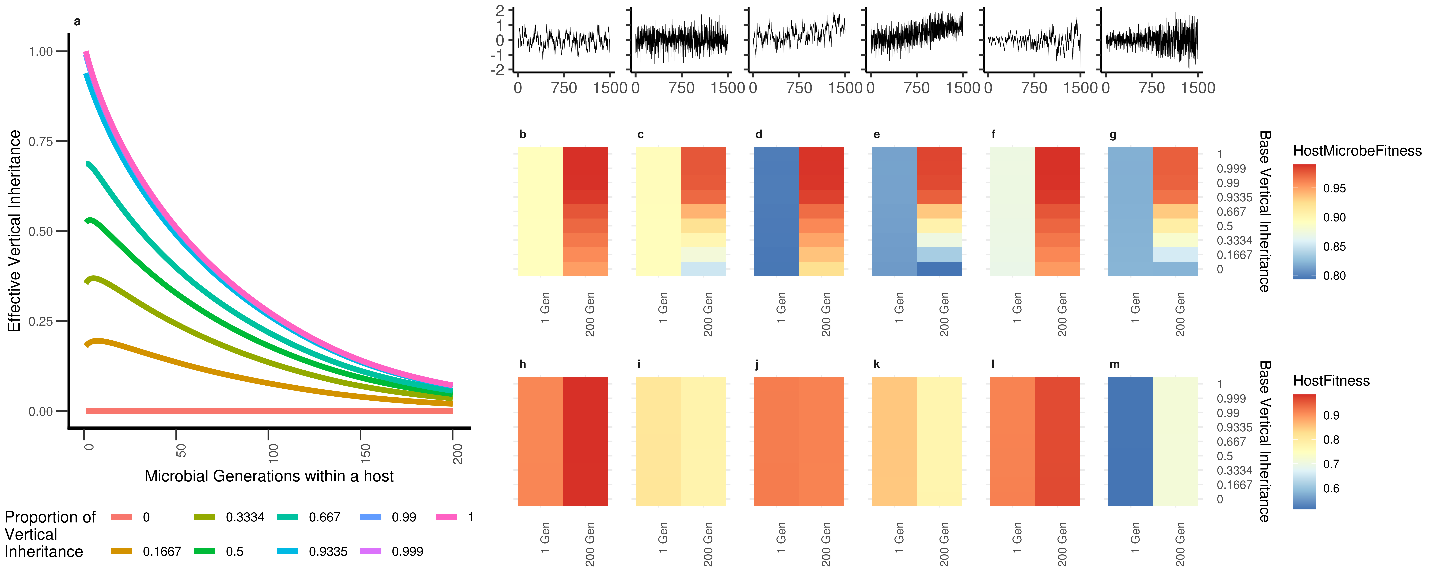
Supp Fig. 14: Patterns of decay in effective vertical inheritance within a host generation for varying levels of initial vertical acquisition (X) (a). Microbe (b-g) and host (h-m) fitness at host generation 1,500 for either 1 or 200 microbial generations per host generation for effective vertical inheritance at different levels. The effective vertical inheritance is standardized such that “1 gen” was run with effective vertical inheritance of 200 generations found in (a). For example, if vertical inheritance is complete but vertical inheritance at 200 generations was determined as 10%, then we initialized a simulation with a vertical inheritance of 10% for 1 generation – and compared this to a simulation where we allowed the simulation to progress for 200 microbial generations where we initialise the vertical inheritance as 100%.
